# Supplementary material for: Antibody response to SARS-CoV-2 infection in humans: A systematic review
Source: PLoS One. 2020 Dec 31;15(12):e0244126. doi: 10.1371/journal.pone.0244126 (PMC7775097; doi:10.1371/journal.pone.0244126)
Supplement: S1 File — (DOCX) [file pone.0244126.s001.docx]

**S1 FILE**

**S1 APPENDIX: Sample search strategy**

Database(s): Embase 1996 to 2020 Week 17
Search Strategy:

| **#** | **Searches** | **Results** |
| --- | --- | --- |
| 1 | exp coronavirus/ | 12437 |
| 2 | exp Coronavirus Infections/ | 12279 |
| 3 | ((corona* or corono*) adj1 (virus* or viral* or virinae*)).tw,kw. | 551 |
| 4 | (coronavirus* or coronovirus* or coronavirinae* or CoV or HCoV*).tw,kw. | 15440 |
| 5 | ("2019-nCoV" or 2019nCoV or nCoV2019 or "nCoV-2019" or "COVID-19" or COVID19 or "CORVID-19" or CORVID19 or "WN-CoV" or WNCoV or "HCoV-19" or HCoV19 or "2019 novel*" or Ncov or "n-cov" or "SARS-CoV-2" or "SARSCoV-2" or "SARSCoV2" or "SARS-CoV-2" or SARSCov19 or "SARS-Cov19" or "SARSCov-19" or "SARS-Cov-19" or Ncovor or Ncorona* or Ncorono* or NcovWuhan* or NcovHubei* or NcovChina* or NcovChinese* or SARS2 or "SARS-2" or SARScoronavirus2 or "SARS-coronavirus-2" or "SARScoronavirus 2" or "SARS coronavirus2" or SARScoronovirus2 or "SARS-coronovirus-2" or "SARScoronovirus 2" or "SARS coronovirus2").tw,kw. | 4488 |
| 6 | (((respiratory* adj2 (symptom* or disease* or illness* or condition*)) or "seafood market*" or "food market*" or pneumonia*) adj10 (Wuhan* or Hubei* or China* or Chinese* or Huanan*)).tw,kw. | 1738 |
| 7 | ((outbreak* or wildlife* or pandemic* or epidemic*) adj1 (Wuhan* or Hubei or China* or Chinese* or Huanan*)).tw,kw. | 91 |
| 8 | ("severe acute respiratory syndrome*" or SARS).tw,kw. | 11647 |
| 9 | or/1-8 | 31091 |
| 10 | exp *serology/ | 16162 |
| 11 | exp *antibody/ | 312568 |
| 12 | exp *antibody response/ | 7205 |
| 13 | exp *seroprevalence/ | 5135 |
| 14 | exp *serum/ | 19113 |
| 15 | exp *assay/ | 93624 |
| 16 | exp *immunity/ | 306567 |
| 17 | exp *diagnosis/ | 1200947 |
| 18 | (sero* or antibod* or serum or sera or test or tests or testing or diagnostic or diagnosis or immunity or ''immune response'').tw,kw. | 6175013 |
| 19 | (assay* adj3 sero*).tw,kw. | 6289 |
| 20 | or/10-19 | 7090637 |
| 21 | 9 and 20 | 10106 |
| 22 | limit 21 to english language | 9349 |
| 23 | limit 22 to dc=20200101-20200331 | 451 |
| 24 | limit 22 to dc=20200401-20200428 | 688 |

**S2 APPENDIX: Data extraction template**

**Supplementary figure B1.** Sample data extraction template as used in the review.

**S3 APPENDIX: Approach to critical appraisal used in the review**

There is a wide range of critical appraisal tools (CAT) available to support evidence assessment and synthesis work in public health and policy, but the applicability of these tools in the context of primary work in immunology is limited by (among other limitations):

- An overwhelming focus on interventional studies, rather than those concerned with exposures;
- A relative absence of tools for critical appraisal of laboratory studies, in particular experimental animal models;
- A relative absence of summative tools that enable critical appraisal of varied study types in a standardised way (rather than application of specific checklists for each study design).
- The time and resource-intensity, which limits scope for reproducible, meaningful, and robust appraisal of evidence in the context of a pandemic in which prodigious volumes of evidence are being generated.

These limitations make it very challenging to identify a coherent approach to critical appraisal that allows for evidence quality to be meaningfully compared across multiple study designs. Preliminary scoping work for this review suggested a tendency in the field of immunology to publication of narrative reviews (rather than conventional systematic reviews) without clear description of study selection criteria or critical appraisal methods. For the purposes of this review, we sought to identify a CAT that:

- Was closely related to an existing, validated tool so that it can be presented as a modification of that tool without the need for new and extended validation work;
- Was low burden and quick to use given required speed of turn-around;
- Facilitated high-level comparison of quality across diverse study designs for reporting purposes;
- Would be rapidly interpretable (ideally in the form of a traffic light system or similar) for time-pressed report readers.

Following a review of existing, generic CATs, we selected the Public Health Ontario MetaQAT 1.0 as our base tool ^13^. This is a qualitative critical appraisal tool based around 4 dimensions (relevance, reliability, validity, applicability of research findings). We made a modification to this tool to include additional domains for publication type (pre-print, peer-reviewed etc) and for assay type and validity. Aggregate assessments of evidence strength against the review’s overarching research questions were also made.

**Supplementary figure C1** shows the tool developed from this process. With the exception of the “publication type” domain, all questions required list-based responses in the “assessment” column – i.e. “Yes”, “No” or “Unsure”. The “Additional comments” was used to provide more detailed qualitative appraisal information, principally to address the prompt questions listed in the “Notes/interpretation” column.

**Supplementary figure C1.** Sample MetaQAT template including prompt questions, and weightings applied to each of the main question domains in the tool for the purpose of quality scoring.

**S4 APPENDIX: Assays used in studies on antibody response**

A range of assays were used in included studies (**supplementary table 1**), although in the majority of cases it was not stated how the assay had been validated internally, or the nature of third party controls that were employed as part of the quality control process. In around half of studies, it was not stated whether commercial assays were performed according to the manufacturer's instructions. Included studies used a range of methods for quantification of neutralising ability, based either on plaque reduction neutralisation tests (PRNT) or microneutralisation tests (MN). In general, EC50 or IC50 were recorded using a range of end points such as cytopathic effect or luciferase activity.

Assays for neutralising antibodies were generally established in house rather than sourced from commercial suppliers and the detailed analysis of these methods was beyond the scope of this review. One study directly compared MN and PRNT neutralisation assays, finding PRNT more sensitive in a small cohort ^73^. Both types of neutralising antibody assays have been employed using either authentic virus or pseudoviral particles in which SARS-CoV-2 S protein has been incorporated into a low pathogenicity, virus-like particle capable of only a single infection cycle. There was no clear predominance of use of either substrate. Two studies suggested that neutralisation sensitivity was lower with primary SARS-CoV-2 than pseudovirus ^85,105^, although this finding was not reproduced in another study ^68^.

| ***Assay*** | ***Number of studies in which assay type was used*** | ***Number in which validation status was reported*** | ***Number in which use as per manufacturer’s instructions was reported*** |
| --- | --- | --- | --- |
| ELISA | 43 | 23 | 28 |
| CLIA/CMIA | 27 | 8 | 14 |
| LFIA | 11 | 8 | 7 |
| CGIA | 4 | 1 | 1 |
| Multiplex | 4 | 3 | 4 |
| LIPS | 3 | 3 | 3 |
| EIA | 3 | 2 | 2 |
| IFA | 3 | 2 | 2 |
| Other | 3 | 2 | 2 |
| In house or commercial assay unable to characterise | 5 | 4 | 3 |
| Assay not described | 8 | n/a | n/a |
| **Total** | **114** | **56** | **64** |

**Supplementary Table 1.** Summary of assays used for serology measurements in included studies by type, reporting of validation status and according to whether use was in accordance with manufacturer’s instructions.

**S5 APPENDIX: FULL LIST OF INCLUDED STUDIES**

| **Paper** | **Country** | **Publication type** | **Study design** | **Population** | **Relevant Outcomes** | **Measurement** | **Findings** | **Quality Assessment** |
| --- | --- | --- | --- | --- | --- | --- | --- | --- |
| Adams, E et al.^58^ | UK | Pre-print | Case control | Mixed hospitalised and community participants 40 cases, 142 controls | IgM and IgG kinetics including titre | ELISA (anti S) (in house) and IgM, IgG and total Ab LFIA (9 commercial assays) | 85% of 40 RT-PCR-confirmed positive patients had IgG detected by ELISA, including 100% patients tested ≥10 days after symptom onset. IgG antibody titres rising over the first 3 weeks from symptom onset, and fell during the second month after symptom onset. No temporal association between IgM titres and time since symptom onset. No evidence that SARS-2-CoV severity, need for hospital admission or patient age were associated with IgG or IgM titres in multivariable models. | Selection of participants not clearly described  Validation of ELISA assay not described |
| Agarwal, V et al.^27^ | USA | Pre-print | Cohort | Hospitalised participants 81 cases | IgG kinetics | Not described | Mean upper bound of time to IgG seroconversion of 38.1 days Data suggests COVID-19 patients potentially continue to shed viral RNA while generating IgG antibodies | Convenience sample with selection poorly described  Incomplete description of study participants  Assay not described, unclear if or how validated |
| Anderson et al.^8^ | Singapore | Peer reviewed paper | Case series | Hospitalised participants 7 cases (SARS-CoV-2) 12 cases (SARS-CoV) | Cross neutralisation ability (SARS-CoV) | ELISA (anti S) (in house) and IgM, IgG and total Ab LFIA (9 commercial assays) | SARS-CoV patient sera contains neutralising antibodies 9-17 years post infection SARS-CoV patient sera has very limited neutralisation ability against SARS-CoV-2 | Very limited detail provided in report limits interpretation of findings  Lack of detail on selection of participants introduces risk of selection bias and lack of control limits generalisability of findings  Assays validated |
| Bao et al.^130^ | Lab or animal based | Pre-print | Basic Science | Animal study (rhesus macaques) | Specific antibody titre following challenge, neutralising antibody titre, protection against reinfection | ELISA (anti S) Neutralising antibody observed by cytopathic effect | SARS-CoV-2 antibody gradually increased to peak 21 days post disease onset 4/4 monkeys showed evidence of neutralising antibody No recurrence of COVID-19 in rechallenged monkeys following primary infection | Methodology clearly described |
| Bao et al.^83^ | Multiple populations | Pre-print | Systematic review with meta-analysis | Mixed hospitalised and community participants 5,912 cases Mean age 54 52% male | IgA, IgM and IgG kinetics including titre T lymphocyte profile | Not described | Severe cases had lower levels of CD4 T cells and CD8 T cells  Compared with the non-severe group, severe cases had lower IgM with no significant difference in other antibodies | Well-conducted review with large number of studies |
| Benotmane et al.^56^ | France | Pre-print | Cohort | Hospitalised participants (kidney transplant recipients) 40 cases Median age 63 75% male | IgM and IgG kinetics including titre | ELISA (anti N and anti S IgM and IgG) (DIA.PRO Diagnostic) | All cases developed antibodies by the second week after symptom onset that persisted for two months. A stable titer of IgG antibodies was maintained until day 59, suggesting persistence for at least until two months IgM and IgG antibody level and delays in seroconversion were not correlated with COVID-19 severity | Selection of sample prone to bias and no controls used  Statistical analysis limited and sample size unclear at different points of study  Assay appears to be validated |
| Borremans et al.^22^ | Multiple populations | Pre-print | Systematic review with meta-analysis | Mixed hospitalised and community participants 516 participants | IgM and IgG kinetics Neutralising antibody detection | ELISA, MCLIA and LFIA PRNT80 | Mean IgG seroconversion time is 13.3 days post symptom onset with ELISA-NP and 12.6 days for IgM using ELISA- S with similar values when using MCLIA, and wide variation between participants. Disease severity does not significantly affect seroconversion time for IgM or for IgG  Maximum probability of IgG detection increases to days 25-27 up to at least 60 days (end of follow up in included studies). Maximum detection probability of IgM peak near 90% around day 25 then decreases.  Detection probability for nAb rapidly rises to near 100%, where it remains up to the last time available in the dataset (29 days), and detection probability does not differ significantly with severity | Well conducted review with a large sample, although lack of clarity in individual study settings and assay validation |
| Brocato et al.^157^ | Lab or animal based | Pre-print | Basic Science | Animal study (syrian hamsters) 30 hamsters | Neutralising antibody titre, protection against reinfection | PRNT80 | Non-immunocompromised hamsters developed a robust neutralizing antibody response compared to immunocompromised hamsters.   Previously infected hamsters who tested positive for neutralising antibodies were re-challenged and none exhibited clinical signs of COVID-19 infection  Hamsters treated with mAbs targeting the S protein of SARS CoV2 did not exhibit clinical signs of COVID-19 infection | Small sample limits findings although controls used  Unclear how assays validated |
| Brochot et al.^126^ | France | Pre-print | Cohort | Hospitalised participants 30 cases Median age 77 66% male | Ab kinetics Neutralising antibody titre | ELISA (anti N, anti RBD, anti S1 and anti S2) (in house) Commercial neutralisation assay | Antibodies targeting the N protein and the RBD were the earliest detected, detectable in all cases after 13 days and anti S2 by 15 days, anti S1 were detected last and not detected in all cases  Significant differences observed between mild and severe disease patients for anti-S1, anti-S2 and anti-N antibody levels, after eight days. Slight difference in anti-N antibody levels according to sex from 14 days. Significant difference in anti-S1 and anti-S2 antibodies according to age between 8 and 14 days  NAb kinetics correlate with production of antibodies targeting the S1, S2 and RBD. NAbs detected in all hospitalised cases by 15 days. NAb titres increased from 1 week and plateau 1 week later, although titers were variable between cases. NAb titre dropped to low or undetectable level in most samples beyond 40 days. Significantly higher NAb titres observed in severe cases and in females. | Methodology generally clearly described although lack of clarity on selection of participants  Risk of bias due to lack of clarity on inclusion and exclusion criteria and relatively small sample  Assays appear to be validated although validated not clearly described |
| Brotons et al.^87^ | Spain | Pre-print | Cohort | Community participants 311 asymptomatic cases Mean age 44 55% female  634 symptomatic cases Mean age 47 58% female | IgG and IgM seroprevalence | LFIA (IgG and IgM) | Reports seroprevalence in sample  Logistic regression concludes seropositive individuals more likely to have symptoms | Well reported study with selection of participants and controls described  Low response rate introduces risk of bias  Assay reported as not validated |
| Brouwer et al.^112^ | Lab or animal based | Peer reviewed paper | Basic Science | 3 participants | Detection of neutralising antobodies and neutralising ability | ELISA (anti S) Pseudovirus and live virus neutralisation assays (not detailed) | Strong and specific B cell responses detected including memory and antibody producing B cells in response to infection. Strongest response in individual with most severe clinical disease.  NAbs isolated which targeted a diverse range of S protein antigenic sites, including both RBD and non-RBD epitopes and NAbs targeting multiple sites. Potent neutralisers of live virus identified. Extensive affinity maturation not required for potent neutralisation. | Sample selection not described introducing risk of bias  Unclear if or how assays validated |
| Bryan et al.^155^ | USA | Pre-print | Cohort | Hospitalised participants 245 cases 60% male | Correlates of IgG detection | ELISA (anti N) (Abbott Architect) | IgG inversely correlated with viral load  33% of participants seroconverted prior to admission  Seroconversion on admission trended towards lower mortality, although not significant | Methodology clearly described  Possibility for selection bias in selection of sample, and lack of clarity in comparator group  Assays validated |
| Buntinx et al.^21^ | Belgium | Pre-print | Cohort | Community participants 201 cases | IgG and IgM seroprevalence | LFIA (IgG and IgM) | No association was found between either IgM or IgG and gender or age, or presence of symptoms.  Of 42 participants positive with PCR, 19 (45%) also had antibodies (IgM/IgG +). Of 134 PCR participants who were negative with PCR, 15 (11%) had antibodies (IgM/IgG +). Of 9 participants tested for antibodies between day 11 and 14 after the positive PCR test, positive IgM results were detected on day 12 and 13 after the positive PCR test, positive IgG results on day 11, 12, 13 and 14 | Methodology and limitations clearly described, although participant characteristics lack clarity  Small sample and lackof characteristics limits generalisability  Assays validated |
| Burbelo et al.^85^ | USA | Peer reviewed paper | Cohort | Hospitalised participants 35 cases, 32 controls Median age 44 87% male | Ab kinetics | LIPS (anti N and anti S) (Sure Screen Diagnostic) | Antibodies against the N and S proteins appeared day 8 and day 14 after the onset of symptoms for a group of 10 cases  Antibody profiles in 3 immunocompromised patients showed more blunted responses | Lack of clarity in selection of participants introduces risk of selection bias  Although controls used, very small sample limits strength of findings and generalisability  Assays validated |
| Bwire et al.^104^ | Multiple populations | Pre-print | Systematic review with meta-analysis | Infants born to CVOVID-19 positive mothers 2 studies included in quantitative synthesis | IgG and IgM kinetics including titre | Not fully described | Median antibody levels in infants born to COVID-19 positive mothers was 75.49AU/mL and 3.79AU/mL for anti-SARS-CoV-2 IgG and IgM, respectively, suggesting passive immunity | Well described review methodology although limited scope |
| Candel Gonzalez et al.^68^ | Spain | Peer reviewed paper | Cohort | Hospitalised participants 35 cases, 5 controls Mean age 57 56% female | IgM and IgG kinetics | LFIA (IgG and IgM) (Autobio Diagnostics Co) | All SARS-CoV-2 positive cases showed corresponding IgG positivity (controls negative) after an average of 28 days from symptom onset. 74% of cases also presented with positive IgM. IgG appeared around day 20 in mild cases and slightly later in severe cases.  IgM lasted longer in severe cases compared to moderate cases but was not observed beyond day 25 in mild moderate cases. | Lack of clarity in sampling methods, particularly serial intervals  Small sample and lack of clarity on characteristics limits generalisability  Detail provided on assay validation |
| Cao et al.^143^ | China | Peer reviewed paper | Basic Science | Hospitalised participants 60 cases Average age and sex not described | Detection of neutralising antibodies and neutralising ability | PRNT and CPE neutralisation assays | 14 potent neutralizing mAbs were identified the most potent of which exhibited an IC50 of 1.2 and 15 ng/mL against pseudotyped and authentic SARS-CoV-2  Prophylactic efficacy demonstrated in mice | Methodology clearly described  Small sample and limited number of controls  Unclear how assay validated |
| Carsetti et al.^69^ | Italy | Pre-print | Cohort | Mixed hospitalised and community participants 64 cases and contacts Among cases mean age 65 (severe) 55 (mild) Among cases 60% male | IgG and IgA kinetics | ELISA (anti S1 IgG and IgA) (Euroimmun) | Higher levels and persistent levels of IgA and IgG, produced relatively late in infection, associated with severe disease  High levels of S1-specific IgA early after diagnosis, rapidly declining and becoming undetectable after 5-7 weeks in asymptomatic cases. Cases with mild disease have IgG and IgA increasing later and remaining relatively low | Selection of sample not described, including for inclusion in subsets, and length of follow up unclear  Assay validated and used according to manufacturers instructions |
| Cervia et al.^78^ | Switzerland | Pre-print | Cohort | Mixed hospitalised and community participants 56 cases Median age 61 55% male  109 exposed contacts Median age 36 77% female | IgG and IgA kinetics including titres | ELISA (anti S1 IgG and IgA) (Euroimmun) | In mild cases, SARS-CoV-2 IgA became positive 8 days after symptom onset and was often transient, IgG levels remained negative or reached positive values 9–10 days after symptom onset. IgA increased slightly as a function of disease duration and IgG increased moderately.  In severe cases, higher IgA and IgG correlatedwith disease duration. Very high IgA correlated with severe acute respiratory distress syndrome (ARDS). 15–20% of S protein-seronegative individuals had detectable S protein-specific IgA antibodies at several mucosal sites. IgA levels inversely correlated with patient age, suggesting increased mucosal antibody responses in younger SARS-CoV-2-exposed individuals. Severe cases had higher serum titers of IgA and IgG.  No overall association between antibody response and age. | Methodology generally clearly described although lack of clarity on treatment of comparator groups  Controls not included and statistical analysis rigorous, although not always clear  Assays appear to be validated |
| Chandrashekar et al.^128^ | Lab or animal based | Peer reviewed paper | Basic Science | Animal study (rhesus macaques) | Neutralising antibody titre, protection against reinfection | ELISA (anti S IgG) (in house) Pseudovirus and live virus luciferase neutralization assays | All 9 macaques developed binding antibody responses to the SARS-CoV-2 S protein and NAb responses using both a pseudovirus neutraliza-tion assay and a live virus neutralization assay. NAb titers of approximately 100 were observed in all animals on day 35. Antibody responses of multiple subclasses were observed against RBD, S and N.  After rechallenge very limited viral RNA was observed in BAL on day 1 following rechallenge in 3 animals, with no viral RNA detected at subsequent timepoints. High levels of viral RNA were observed in concurrently challenged naïve animals. Little or no clinical disease was observed in the animals following rechallenge. NAb titers were markedly higher on day 14 following rechallenge compared with day 14 following primary challenge | Methodology well described and methods appear to be appropriate including use of controls, although small study  Assays validated |
| Chen et al.^98^ | China | Peer reviewed paper | Case series | Hospitalised participants Children 20 adult controls Median age 14.5 6 cases male | IgG and IgM titres | Not described | No significant difference between levels of antibodies between children and adults: children 1.22 +/-0.39 g/L IgM, 10.86 +/-1.44 g/L IgG  Children had higher total T cell, CD8+T cell and B cell than adults | Lack of clarity in selection of participants, particularly for serial measurements  Comparison sample used although not controlled and lack of clarity limits interpretation of findings  Assays validated and used according to manufacturers instructions |
| Chen et al.^144^ | China | Peer reviewed paper | Basic Science | Hospitalised participants 26 cases | IgG titres | ELISA (anti S1 and anti RBD) | 23 of 26 cases produces high titers of S1-specific IgG antibodies, 3 mounted relatively lower anti-S1 IgG responses, only 3 patients showed effective blockade of SARS-CoV-2 RBD binding to hACE2. SARS Cov-2 RBD-specific IgG antibodies were present in sera of all patients by ELISA | Lack of clarity in selection of participants, particularly for serial measurements  Comparison sample used although not controlled and lack of clarity limits interpretation of findings  Assays validated and used according to manufacturers instructions |
| Chen et al.^124^ | China | Peer reviewed paper | Cohort | Community paticipants 105 healthcare worker contacts of cases Median age 30 79% female | IgG and IgM seroprevalence and neutralising ability | EIA (anti N and anti RBD IgG and IgM) (in house) | 19 serum samples showed various degree of neutralization capability, in which from 42% to 99% of SARS-CoV-2 pseudovirus was neutralized. The percentage of neutralization was correlated with anti-NP IgG and anti-RBD IgG 8 out of 19 serum samples showed neutralization percentage of over 50%. | Methodology and population well described, and methods appear appropriate although small sample  Assays validated |
| Chi et al.^115^ | China | Peer reviewed paper | Basic Science | Hospitalised participants 10 cases Age range 23 - 53 | Detection of neutralising antibodies and neutralising ability | ELISA (anti S and anti N)  Pseudovirus and authentic virus luciferase neutralisation assays | Of 35 identified mAbs, only 3 neutralised authentic SARS-CoV-2. Neutralizing ability of plasma against authentic and pseudovirus were correlated although some mAbs neutralised pseudovirus but not authentic virus. On mAb had high neutralising potency but did not bind to RBD. | Selection of sample not described, and very small study although exploratoy desgin  Assay use well described altough unclear if validated |
| Choe et al.^117^ | South Korea | Peer reviewed paper | Case control | Hospitalised participants 7 asymptomatic cases Median age 25 72% male  17 symptomatic cases 7 subtle (43%M, median age 53y), 4 apparent but mild (75%M, median age 59y), 6 apparent and severe(83%M, median age 75y) | IgG titre (by optical density) Detection of neutralising antibodies and neutralising ability | ELISA (anti S1 IgG) (Euroimmun) | 71% of asymptomatic cases seropositive. Higher optical density value in patients with pneumonia; titers correlated with disease severity. All patients showed neutralizing antibody response.   Geometric mean titer 78 in asymptomatic patients (n = 7), 256 in patients with subtle pneumonia (n = 4), and 3,158 in patients with apparent pneumonia (n = 8; 4 mild and 4 severe cases). | Very small sample and selection not clearly described  Unclear if or how assay validated  Lack of detail in reporting limits interpretation of findings |
| Cohen et al.^99^ | France | Pre-print | Case control | Community participants 605 cases (children) Mean age 4.9 53% asymptomatic, 47% paucisymptomatic | IgG and IgM seroprevalence | LFIA (IgG and IgM) (Biosynex) | Only 3/605 children were RT-PCR–positive without any antibody response. The frequency of positivity on RT-PCR for SARS-CoV-2 was significantly higher in children with positive serology than those with negative serology | Well conducted study with large sample and robust statistical analysis  Assay validated |
| Dahlke et al.^71^ | Germany | Pre-print | Case series | Hospitalised participants 4 cases and 1 control | SARS-CoV-2 proteome, host B cell immunophenotyping and IgG, IgM and IgA serum antibody interactions | Peptide microarrays | Mild cases displayed stronger IgA response day 3-4 that decreased during the course of disease 7 – 14 days later. IgG response similar but less pronounced trend and appears more persistent. The number of reactive IgG peptides lower in the early acute phase in comparison to IgA.  Data showed generally more IgA than IgG or IgM epitopes, with most epitopes located in the S and N protein.  Seroconversion occurred at day 7 post onset of symptoms in 50% of infected individuals. | Lack of clarity in selection of partipants introduces risk of selection bias  Very small sample  Unclear if or how assay validated |
| Danh et al.^106^ | Multiple populations | Pre-print | Basic Science | 37 convalescent plasma samples | Neutralising antibody detection | In house neutralisation assay | NAb detectable at 10 days after onset of symptoms and continue to rise, plateauing after 18 days.  S1 proteins showed stronger NAb signals. | Methodology clearly described  Small convenience sample introduces risk of bias although controls included  Assay validated |
| De Vriese et al.^57^ | Belgium | Peer reviewed paper | Case series | Hospitalised participants 7 cases | IgG kinetics | ELISA (anti N IgG) (NovaLisa) | Overall IgG seroconversion rate was 100% and by the second week after symptom onset. Antibody titer plateaued during the third week.  Anti-SARS-CoV-2 IgG antibodies coexistied with virus suggesting these antibodies are not neutralizing | Limited description of methodology limits interpretation of findings  Lack of detail on selection of participants and lack of controls limits generalisability  Assay validated |
| Deeks et al.^175^ | Multiple populations | Peer reviewed paper | Systematic review with meta-analysis | Mixed hospitalised and commnity participants 8,526 cases from 54 studies | Sensitivity and specificity of antibody assays | ELISA (anti N IgG) (NovaLisa) | Pooled results for IgG, IgM, IgA, total antibodies and IgG/IgM all showed low sensitivity during the first week since onset of symptoms (all less than 30.1%), rising in the second week and reaching their highest values in the third week. The combination of IgG/IgM had a sensitivity of 30.1% for 1 to 7 days, 72.2% for 8 to 14 days, 91.4% for 15 to 21 days.  Estimates of accuracy beyond three weeks are based on smaller sample sizes and fewer studies. For 21 to 35 days, pooled sensitivities for IgG/IgM were 96.0%. There are insufficient studies to estimate sensitivity of tests beyond 35 days post-symptom onset.  Summary specificities (provided in 35 studies) exceeded 98% for all target antibodies with confidence intervals no more than 2 percentage points wide. False-positive results were more common where COVID-19 had been suspected and ruled out, but numbers were small and the diference was within the range expected by chance. | Well conducted review with appropriate statistical analysis  Detail on assay performance provided |
| den Hartog et al.^19^ | Netherlands | Pre-print | Cohort | Mixed hospitalised and commnity participants 115 cases, 334 controls | IgG kinetics including titre | Multiplex assay (anti S1, anti RBD and anti N) | Hospitalised cases developed higher IgG concentrations and the rate of IgG production increased faster compared to non-hospitalised cases.   Hospitalised cases seroconverted around day 10 of disease onset and reached a plateau after two weeks (25 days for non-hospitalised cases) | Sample selection not well described including size of subgroups introduces the risk of bias  Detail on assay validation provided |
| Dingens et al.^100^ | USA | Pre-print | Case series | Mixed hospitalised and community participants 10 cases of 1,076 total participants Children 50% female | Antibody kinetics Neutralising antibody detection and neutralising ability | ELISA (anti S and anti RBD IgG)  Pseudovirus neutralisation assay | Demographic and clinical data had no detected associations with seropositivity  Eight of 10 seropositive children neutralized virus at a >1:25 dilution with neutralization correlating with RBD and spike binding as measured in the ELISAs. Two children had very high neutralizing activity, including one with a potency that exceeded the limit of our assay (>1:18,000 dilution). The two children without neutralizing activity had not experienced symptoms | Methodology and limitations clearly described  Sample selection vulnerable to bias and very small sample limits generalisability  Assays validated |
| Dobi et al.^74^ | Reunion | Pre-print | Cohort | Hospitalised participants 20 cases | IgG and IgM kinetics | ELISA and IFA (IgG and IgM) | IgM and IgG detected early during the course of the disease (5-7 days post-first symptoms), significant decrease of IgG levels in severecases - for these cases seroconversion for IgG and IgM occurred simultaneously and antibody titers plateaued within 6 days after seroconversion.  In mild cases a steady rise in specific IgG was observed. Both responses (IgM and IgG) were initially against N and spreading to other S1 and S2 | Lack of clarity in description of methods limits interpretation  Unclear how participants selected and results not well described, intriducing potential for bias  Unclear how assays used or if or how validated |
| Du et al.^47^ | China | Peer reviewed paper | Case series | Hospitalised participants 60 cases | IgG and IgM kinetics including titres | Not described | All cases tested positive for IgG while 13 tested negative for immunoglobulin M (IgM), with the immunoglobulin G (IgG) titer being greater than the IgM titer.  IgM and IgG titers in 10 cases were tested twice (1 week apart, i.e. 7-8 weeks since symptom onset); both titres showed a decrease, with the IgG titer still remaining greater than the IgM titer. | Validation of assay not described |
| Du et al.^101^ | China | Peer reviewed paper | Cohort | Hospitalised participants 182 cases (children) Median age 6 66% male | IgG and IgE titre | Not described | IgG significantly lower in those with pneumonia than in those without  Serum total IgE level of allergic children was higher than that of non-allergic individuals, and allergic children had greater percentage of increased IgE | Lack of clarity on methods for testing and time points  Risk of bias in inclusion of participants  Assay validation not described |
| Duan et al.^75^ | China | Pre-print | Case control | Hospitalised participants 616 cases Median age 64 57.5% female | Correlates of antibody detection | Not described | Compared with confirmed cases (antibody positive),, antibody negative probable cases were younger, had higher lymphocytes and similar symptom and comorbidity profile | Lack of clarity of description of methodology limits interpretation, including the size of the sample and comparator group  Comparator group characteristics differed at baseline introducing bias  Assay validation not described |
| Edouard et al.^20^ | France | Pre-print | Cohort | Hospitalised participants 888 cases Median age 45 54% female | IgG and IgM kinetics including titre | IFA (in house) | Seroprevalence 47% after 15 days after disease onset. Seroconversions of specific IgM and IgG antibodies were observed as early as day four after the onset of symptoms  Severe cases developed a serological response in most cases (and all patients who died) that was characterised by high levels of IgG  Significant higher IgG titre in cases with a poor clinical outcome compared to cases with good outcome | Lack of clarity in selection of participants and description of sampling from them  Methodology appears appropriate and relatively large sample size  Assays validated |
| Ejemel et al.^152^ | Lab or animal based | Pre-Print | Basic Science | 36 serum samples | Detection of neutralising antibodies and neutralising ability | ELISA (anti S) nautralising assay not described | IgA mAb neutralizes via competing S protein binding to hACE2 receptors. IgG weakly neutralizes SARS-CoV-2 while its IgA1 isotype variant and its dimeric form showed significantly enhanced neutralization potency | Lack of clarity in description of methods although methods appear largely appropriate  Assay validation not described |
| Espejo et al.^132^ | Multiple populations | Peer reviewed paper | Systematic review without meta-analysis | 55 studies included | Sensitivity and specificity of antibody assays | ELISA, CLIA and LFIA | Total antibody or combined IgG/IgM detection offered the highest sensitivity of detection  Conflicting evidence regarding whether antibody titres correlated with clinical severity  Preliminary investigations indicated some immunoassays may be a surrogate for the prediction of neutralizing antibody titers and the selection of recovered patients for convalescent serum donation | Inclusion criteria for studies not described and quality assessment not used  Detailed description of assay validation provided |
| Fafi-Kremer et al.^108^ | France | Pre-Print | Cohort | Community participants 160 cases Median age 32 69% female | Antibody kinetics Neutralising antibody detection | Anti RBD rapid test (Biosynex) and anti S flow cytometry | Antibodies against SARS-CoV-2 were detected in virtually all hospital staffsampled from 13 days after the onset of COVID-19 symptoms.   The neutralizing activity of the antibodies increased over time. Neutralizing ID50≥100 were found in 91% of the individuals, reaching 97% four weeks after the onset of symptoms.  Individuals with factors associated with more severe disease (e.g., male sex, high body mass index and high blood pressure), were more likely to have high titers of neutralizing antibodies compared to others. | Risk of selection bias, particularly between groups and no controls described  Assay appears to be validated |
| Farrera et al.^153^ | Switzerland | Pre-print | Basic Science | Hospitalised participants 12 cases, 6 controls | Antibody epitope mapping | ELISA (anti S1 IgG) (Euroimmun) Peptide array | 3 linear epitopes most abundantly detected (SARS-CoV-2 S protein) were: 655-672, 787-822, and 1147-1158. None of these epitopes was singularly detected in all the positive samples tested, but each is detected in >40% of positive cases.   Antibodies binding to protease cleavage sites were identified from COVID-19 patients, raising the possibility that other mechanisms than blocking the RBD-ACE2 interaction could be harnessed for neutralization. Blocking proteolytic cleavage could be important to reduce antibody-dependent enhancement of viral entry, a key feature for vaccine development | Methodology generally clearly described although lack of clarity on selection of participants  Although small sample, appears appriate for study aims  Assays validated |
| Favara et al.^88^ | UK | Pre-print | Cohort | Community participants (healthcare workers) 70 cases Median age 41 87% female | IgG seroprevalence and correlates | LFIA (anti N and anti S IgG and IgM) (Abbexa) Multiplex assay (anti S and anti N IgG) | IgG positive cases had a 3.6 times higher odds of having previously had symptoms  13% of cases who reported no prior symptoms were IgG positive, 28.6% amongst those asymptomatic who were exposured to a suspected case in their household  12.5% of participants with chronic underlying health conditions had positive antibodies in contrast to 24.1% of those without. | Small sample size, including small subgroups  Limited statistical analysis  Assays validated |
| Fox et al.^105^ | USA | Pre-print | Case series | Hospitalised participants 15 cases | IgG, IgM and IgA titres (in breast milk) | ELISA | Most milk (80%) obtained post-COVID-19-recovery exhibits IgA reactivity to RBD  Mean OD values of undiluted milk in COVID-19-recovered group were significantly greater for IgA, secretory-type Abs, and IgG, but not for IgM  No correlation between the reactivities of Ab subclasses, with the exception of IgM v IgG, which were found to correlate | Lack of clarity in selection of sample intoduces risk of selection bias  Small sample limits generalisability although controls used and statistical analysis appropriate to identify significant results  Assay validated |
| Fung et al.^28^ | USA | Peer reviewed paper | Case series | Mixed hospitalised and community participants 10 cases (solid organ transplant recipients) Median age 57 60% male | IgG kinetics | CLIA (anti N IgG) (Abbott) | 100% of hospitalised cases had positive SARS-CoV-2 IgG, 6 observed to seroconvert between day 6 and day 28 after symptom onset | Very small sample and lack of controls limits generalisability of findings  Assay validated |
| Galson et al.^154^ | UK | Pre-print | Basic Science | Hospitalised participants 19 cases Mean age 50 68% male | B cell receptor heavy chain sequencing | BCR sequencing | Stereotypical naïve immune response to SARS-CoV-2 which is consistent across patients and maybe a positive indicator of disease outcome.  SARS-CoV-2 infection stimulates both naïve and memory responses - Clonal expansion of the B cell memory response is also observed and may be the result of memory bystander effects.  Sequence convergence can be used to identify putative SARS-CoV-2 specific antibodies. BCR clonotype sequence convergence signatures are shared between different COVID-19 studies in different locations and from different anatomical sites: the BCR repertoires of the COVID-19 patients were significantly  more diverse than the BCR repertoires of the healthy controls; this increase in diversity was positively correlated with an increased proportion of unmutated sequences | Unclear exclusion and inclusion criteria without controls, although exploratory design  Very small sample  Assays appear to be validated although not clearly described |
| Garcia-Basteiro et al.^94^ | Spain | Pre-print | Case control | Community participants 578 healthcare workers | IgG, IgM and IgA kinetics | Multiplex assay | Among seropositive cases no statistically significant associations of antibody levels with sex. IgM levels positively correlated with age. IgA levels higher in participants reporting COVID-19-compatible symptoms in the previous month than in those asymptomatic. Duration of symptoms greater than 10 days associated with higher IgM levels  Antibodies detected in individuals with 6 or more days between symptoms onset and recruitment for IgA and later for IgM and IgG with no seropositive results detected among individuals with symptoms onset less than 6 days prior to recruitment.  Antibody levels increased and peaked between day 20 and 25 for IgM and IgG, and a few days earlier for IgA | Well described methodology and approach to statistical analysis  Robust approach to sampling  Assay validated |
| Giagulli et al.^97^ | Multiple populations | Peer Reviewed paper | Narrative Review | Details of included studies not provided | IgG seroprevalence and correlates | Not described | In a study with n=331, delayed peak of antibody response with a lower generation of effective IgG was found in men compared to women | Non-systematic methodology used, lack of clarity on studies included and quality assessment introduces risk of bias for selection and interpretation of studies |
| Gniffke et al.^29^ | USA | Pre-print | Case control | Hospitalised participants 24 cases Median age 42 Female 71%  30 controls Median age 37 Female 60% | IgG kinetics Neutralising ability | Flow Cytometry (anti S and anti RBD IgG) | Anti S IgG and anti RBD IgG in cases detected at median of 36days following documented SARS-CoV-2 RNA secretions   COVID-19 case samples significantly inhibited binding compared to no plasma and pre-covid plasma. Case samples produced greater inhibition than pre-COVID for both trimer and RBD  Fever associated with increased anti-trimer IgG and inhibition | Unclear inclusion and exclusion criteria with potential for selction bias, although controls used  Small sample with limited range of characteristics  Assay use well described |
| Grzelak et al.^18^ | France | Pre-print | Case control | Mixed hospitalised and community participants 260 cases, 491 controls | Antibody titres Neutralising antibody titres | ELISA (anti N and anti S) and LIPS (anti N and anti S) (in house)  Pseudovirus neutralisation assay | High antibody titers associated with neutralisation activity  In hospitalized patients, seroconversion and neutralisation occurred on 5-14 days post symptom onset  Seropositivity was detected in 29% of pauci-symptomatic individuals within 15 days post-symptoms and 3 % of blood of healthy donors collected in the area of a cluster of COVID cases | Lack of clarity on selection of participants, particularly for neutralising antibody assessment  Small sample and possibility of selection bias limits generalisability of findings  Assay validated |
| Hansen et al.^149^ | Lab or animal based | Peer reviewed paper | Basic Science | 3 human cases and animal model (mice) | Detection of neutralising antibodies and neutralising ability | ELISA VZV-based psuedoparticle assay | All of the detected neutralizing mAbs bound to the RBD of SARS-CoV-2 spike and blocked its ability to interact with ACE2 with double-digit pM IC50s supporting ACE2 blockade as the primary mechanism for neutralization  The antibodies bound specifically and with high affinity to monomeric SARS-COV-2 RBD (KD = 0.56 to 45.2nM) and dimeric SARS-COV-2 RBD (KD = 5.7 to 42.8 pM). While recombinant ACE2 was able to mediate neutralization of the VSV-spike pseudoparticles as previously reported, it was more than 1000-fold less potent than the best neutralizing mAbs.  The IC50s of all Fabs were shifted compared to their parental IgG, indicating that biva-lent binding, cross-linking and steric hindrance might all augment neutralization.  A small subset of antibodies showed significantly higher neutralising potential against non-replicating pVSV-SARS-CoV-2-S- mNeon and replicating VSV-SARS-CoV-2-S virus, with mAbs combinations appearing to have neutralising impact at lower concentrations | Lack of clarity regarding methods including selection of samples  No controls used although exploratory study  Unclear if assays validated |
| Hartman et al.^41^ | Lab or animal based | Pre-print | Basic Science | Animal study (African green monkeys) | IgG and IgM kinetics Neutralising antibody detection | ELISA PRNT80 | All animals seroconverted with IgM and IgG in the second week post exposure and those that were exposed via the mucosal route had higher overall titers than those that received the virus via the aerosol route  Neutralising antibodies detected in samples taken 7-11 days post infection. There were no differences in kinetics or activity between the two exposure routes demonstrating that higher dose received during multi-route mucosal infection neither affected the onset nor strength of the humoral immune response. | Small sample size, no controls used and lacks statistical analysis  Unclear if assays validated |
| Harvala et al.^109^ | UK | Pre-Print | Case series | Hospitalised participants 52 cases 100% male | Detection of neutralising antibodies and neutralising ability | ELISA (anti S1 IgG) (Euroimmun and Fortress) Pseudovirus and live virus microneutralisation assay | Neutralising antibodies detected in 43 of 52 samples using a cut-off titre of 1:20. The highest detectable titre was 1:4096.  Correlation observed between neutralizing antibody titres and reactivity IgG ELISA and between neutralizing antibody titres and IC50 values in pseudotype assay | Small sample with limited range of characteristics and lack of clarity in selection introduces risk of bias  Lack of controls |
| He et al.^80^ | China | Peer reviewed paper | Case control | Hospitalised participants 204 cases | IgG, IgM, IgA and IgE titres | Not described | Higher IgG in severe cases, with lower IgM. IgM and IgA significantly lower in patients with comorbidities | Lack of clarity in selection of sample particularly for longitudinal testing  Very small sample size and lack of control limits generalisability  Validation of assay not described |
| He et al.^50^ | China | Peer reviewed paper | Cohort | Hospitalised participants 169 cases Median age 55 54% male | IgG and IgM kinetics | CGIA (Tangshan) and CLIA (IgG and IgM) (Maccura and Tanjin) | Cumulative positive IgM conversion rate was close to 100% after 3 weeks by both assay methods  After 23 days the cumulative positive conversion rate of IgG reached more than 95%. Regardless of assay, no downward trend in positive rate of IgG detection during the observation period (35 days)  The positive conversion time of IgG is 2 days later than IgM by chemiluminescence method on average, and it is 3 to 5 dayslaterby colloidal gold method. | Unclear how participants and controls were selected  Assays reported as validated, although unclear how controls used or if used according to manufacturers instructions |
| Herroelen et al.^36^ | Belgium | Pre-print | Cohort | Mixed hospitalised and community participants 135 cases and suspected cases | IgG, IgM and IgA kinetics | Multiple ELISAs and LFIAs (Wantai, Orient Gene, Elecsys, Euroimmun, Innovita, Liaison | Lack of seroconversion by any test was seen in 1.4% of hospitalized and 4.7% of paucisymptomatic infections  All tests except Wantai SARS-COV-2 Ab ELISA showed a significantly higher positivity rate between 10 and 20 days post onset as compared to less than 10 days. No significant differences in positivity rates between 10 and 20 and more than 20 days | No inclusion or exclusion criteria described  Limited statistical analysis limits findings  Assay appeears to be validated with controls |
| Hou et al.^53^ | China | Peer Reviewed paper | Cohort | Hospitalised participants 338 cases Mean age 62 50% male | IgG and IgM kinetics | CLIA (IgM and IgG) (YHLO) | The median number of days from symptom onset to antibody detection was not significantly different across the mild, severe and critical groups (20.95 +/- 9.226 days, 21.9 +/- 8.724 days and 20.86 +/- 8.126 days, respectively) .  Severe and critical cases had higher IgM levels than mild cases, whereas IgG in critical cases was lower than those in both mild and severe cases. IgM levels slightly higher in deceased patients than recovered patients, but IgG levels in these groups did not significantly differ.  A longitudinal detection of antibodies revealed that IgM levels decreased rapidly in recovered patients whilst IgG was detectable for longer. IgM level reached its peak after 2 weeks and then decreased rapidly by 3 weeks. IgG was maintained at a high concentration even after 7 weeks. In deceased cases, either IgM levels remained high or both IgM and IgG were undetectable during the disease course. | Large sample including controls, although lack of clarity on selection of controls with potential for bias  Assay validated and used according to manufacturers instructions |
| Houlihan et al.^92^ | UK | Pre-Print | Cohort | Communtiy participants (healthcare workers) 200 participants Median age 34 61% female | Seroprevalence amongst healthcare workers | ELISA | All but one HCW with PCR evidence of infection developed antibodies. Antibodies to the spike protein of SARS-CoV-2 remained detectable over the one month of follow up. There was no evidence of reinfection in those with antibodies at baseline over one month follow up. | Small sample particularly for certain subgroups limits generalisability, and statistical analysis not well described  Risk of bias in selection of participants and accuracy of test results  Assay validated |
| Hu et al.^63^ | China | Pre-print | Cohort | Hospitalised participants 211 cases Median age 47 40% female | IgG and IgM kinetics including titres | MCLIA (IgG and IgM) (Chonqing Bioscience) | Peak of both antibodies on day 19-21, after which IgM gradually decreased while IgG remained at a high level, with a median concentration of 17·38 and 5.59 (IQR 0·73，13·65), respectively. IgG significantly higher in the severe group than in the mild group; IgM level in the two groups not significantly different  Of 74 recovered patients 39 (52·7%) presented with re-detectable positive virus nucleic acid during this period. These patients had significantly lower IgG concentration within 7 days after discharge but the difference in IgM concentration was not significant. The decrease of SARS-CoV-2 IgG and IgM antibodies in 40 recovered patients between two tests within 14 days after discharge with a 7-day interval was not statistically significant but the decrease of IgG and IgM each reached 21·2%  This study found no correlation between IgG and IgM concentration and viral shedding, course and outcome of the disease through a retrospective analysis without the knowledge of SARS-CoV-2 viral load. | Methodology generally well described although lacks clarity on aspects of selection of participants, introducing risk of bias  Large sample size and appropriate statistical analysis conducteed  Assays validated |
| Hu et al.^113^ | China | Pre-print | Basic Science | Hospitalised participants 41 convalescent plasma samples | Detection of neutralising antibodies and neutralising ability | Pseudovirus luciferase neuralisation assay | Among the 41 tested sera, 38 showed neutralizing activities against one or both pseudovirus. Sera from 3 cases high neutralizing activity against one pseudovirus with ID50 ranging from 894 to 1337, but decreased titres against an alternative pseudovirus (alternative pseudoviruses related to identified S-D614 to S-G614). These data indicated that D614G mutation changed the antigenicity of S protein, thereby decreasing neutralization sensitivity to individual convalescent sera. | Lack of inclusion and exclusion criteria, potential for selction bias  Small samplee and lack of statistical analysis limits findings  Assay described with controls used although not clearly validated |
| Huang et al.^38^ | China | Pre-print | Cohort | Hospitalised participants 33 cases Median age 47 52% male | IgG and IgM kinetics | MCLIA (IgG and IgM) (Chonqing Bioscience) | 8 weeks from symptom onset, IgM negative in many of the previously positive patients, and IgG levels remained less than 50% of the peak levels in more than 20% of the patients. In about 40% of the patients, anti-RBD IgG levels increased 4-times higher in convalescence than in acute phase.  The median seroconversion time of anti-S IgM, anti-RBD IgM, and anti-N IgM was 10.5 days, 14 days, and 10 days, respectively. The median seroconversion time of anti-S IgG, anti-RBD IgG, and anti-N IgG was 10 days, 13 days, and 10 days, respectively. SARS-CoV-2 RNA coexisted with antibodies for more than 50 days. Anti-RBD IgM and IgG levels, including anti-RBD IgM levels at presentation and peak time, were significantly higher in viral RNA short persistence patients than in long persistence cases. | Lack of clarity in selection of sample including frequency of sampling  Small sample size and lack of controls limits generalisability of findings  Assay validated and used according to manufacturers instructions |
| Huang et al.^7^ | Multiple populations | Pre-print | Systematic review with meta-analysis | Multiple populations 40 studies in quantitative synthesis | IgG and IgM kinetics and correlates | Mutliple assays including ELISA, IFA, Western blot, HAI and complement fixation, and neutralisation assays | The median time to antibody detection was similar across different antibodies for SARS-CoV-1 (12 days; IQR 8-15.2 days) and SARS-CoV-2 (11 days; IQR 7.25-14 days), but longer for MERS-CoV (16 days; IQR13-19 days). Most long-term studies found that IgG waned over time - Antibody kinetics varied across the severity gradient with longer durations of detectable antibody associated with more severe symptoms.    SARS-CoV-2 is most similar to SARS-CoV-1, harboring sequence homology of 90% in N and 76% in S followed by MERS-CoV (48% and 35%, respectively)  While IgM antibodies appear at the same time in severe and non-severe cases, IgG appears sooner in severe cases. On the other hand, neutralizing antibody titerswere higher in severe cases. Severity appears to be associated with time to detection of IgM in MERS-CoV cases only (2 days longer), and IgG in both MERS-CoV and SARS-CoV-2 (2 to 3 days longer for more severe cases) | Well conducted review although limited description of criteria for inclusion of data from studies and methodology for pooled analysis  Lack of clarity on validation of assays in included studies |
| Hurlburt et al.^150^ | Lab or animal based | Pre-print | Basic Science | 1 convalescent plasma sample | Structural mechanism of RBD/ACE2 receptor blocking | Neutralisation assay | CV30 binds almost exclusively to the concave ACE2 binding epitope (also known as the receptor binding motif (RBM)) of the RBD using all six CDR loops with a total buried surface area of ~1004 Å2, ~750 Å2 from the heavy chain and ~254 Å2 from the kappa chain  The structure reveals that the CV30 epitope overlaps almost completely with the ACE2 epitope. A total of 26 residues of the SARS-CoV-2 RBD interact with hACE2, CV30 binds to 19 of these residues 3) Additionally, the minimal affinity maturation observed 21 days after infection in the VH gene of CV30 showed ~100-500-fold increase in affinity and neutralization potency, indicating that further affinity maturation may increase potency and potential cross-reactivity.  Of the interacting residues from the SARS-CoV-2 RBD, only 16 are conserved in the SARS-CoV S protein RBD, which could explain the lack of cross-reactivityof CV30 to SARS-CoV S1547. Thus,minimal affinity maturation of CV30 significantly impacted the ability of this mAb to neutralize SARS-CoV-2. | Sample source and characteristics not described  Assay described although unclar how validated |
| Imai et al.^131^ | Lab or animal based | Peer reviewed paper | Non-randomised controlled trial | Animal study (syrian hamsters) 8 hamsters | Neutralising antibody titre, protection against reinfection | ELISA Neutralising assay | SARS-CoV-2−infected hamsters mounted neutralizing antibody responses and were protected against subsequent rechallenge with SARS-CoV-2  Passive transfer of convalescent serum to naïve hamsters efficiently suppressed the replication of the virus in the lungs even when the serum was administrated 2 d postinfection of the serum-treated hamsters  Virus titers in the nasal turbinates and lungs of the animals that received postinfection serum on day 1 postinfection were statistically significantly lower than the virus titers in those organs of animals that received normal serum at the corresponding time point postinfectio. No statistically significant differences in the virus titres in the respiratory organs were found between the animals that received postinfection serum and those that received normal serum on day 2 postinfection | Lack of incllusion and exclusion criteria and small sample, although exploratory study  Limited description of assay use |
| Jaaskelainen et al.^35^ | Finland | Peer Reviewed paper | Case control | Hospitalised participants 40 cases median age 56 65% male  37 controls median age 53 60% female | IgG and IgA kinetics | ELISA (anti S1 IgG and IgA) (Euroimmun) | IgA and IgG detected in 13/39 confirmed COVID-19 cases, IgA detected alone in 11/39. Total positive = 24/39  The median time after onset of symptoms was 12 days for detection of IgGs, and 11 days for detection of IgAs   Amongst probable cases, 1/13 postive for IgA and IgG, 1/13 positive for IgG alone and 1/13 for IgA alone  Did not see any clear patterns between the IgG or IgA results and disease severity | Very small sample and risk of bias in selection of participants as recruitment not clearly described  Limited statistical analysis performed  Assay validated |
| Jin et al.^46^ | China | Peer reviewed paper | Case control | Hospitalised participants 43 cases and 33 controls median age 47 | IgG and IgM titres | CLIA (IgG and IgM) | The IgM-positive rate increased slightly initially then decreased over time; in contrast, the IgG-positive rate increased to 100% and was higher than IgM at all times.   Both IgM and IgG levels were not high in the first 5 days following symptom onset. In the COVID-19 group. Median IgM and IgG titers among cases were 12.1AU/mland 132.2AU/ml, respectively.  IgM-positive rate and titer were not significantly different before and after conversion to virus-negative. The IgG-positive rate was up to 90%and also not significantly different before and after conversion to virus-negative. However, the median IgG titer after testing virus-negative was double that before, and the difference was statistically significant. | Generall well described methodology although lack of clarity in selection of participants  Control group used and robust use of comparator group  Assays validated |
| Jin et al.^55^ | China | Peer reviewed paper | Cohort | Hospitalised participants 89 cases Median age 62 55% female | IgG and IgM kinetics including titres | CLIA (IgG and IgM) | Pooled serum IgM was significantly higher in the prolonged shedding groups at weeks 4 and 5 compared with the non-prolonged shedding group  Serum IgM was similar between two groups from week 6 to week 8 after symptom onset. At week 8, serum IgM in both groups declined almost to the reference level  Serum IgM to SARS-CoV-2 persisted at a high level during the acute phase of illness up to week 8. In individuals with prolonged viral RNA shedding, serum IgM was found to be positive at week 6 after symptom onset, which was also consistent with the median time to viral RNA shedding (44 days). Serum IgG persisted at a high level up to 8 weeks in both groups | Limited detail in report due to format limits interpretation of findings  Small sample and unclear how selected  Assay validated and reports used according to manufacturers instructions |
| Ju et al.^145^ | China | Peer Reviewed paper | Basic Science | Hospitalised participants 8 cases, 1 control age range 10-66 | Detection of neutralising antibodies and neutralising ability | ELISA | 206 RBD-specifc monoclonal antibodies isolated. Antibodies were identified with potent anti-SARS-CoV-2 neutralization activity that correlates with their competitive capacity with ACE2 for RBD binding. Many antibodies with high Kd values minimally competed with ACE2, suggesting binding affinity does not predict ACE2 competing capacity  No single or group of antibody families stood out across various patients, suggesting different patients had immunologically distinct responses. Plasma samples from severe demonstrated relatively higher anti-SARS-CoV-2 binding activities than those from mild cases  Neither the anti-SARS-CoV-2 antibodies nor the infected plasma cross-reacted with SARS-CoV or MERS-CoV RBDs, although substantial plasma cross-reactivity to their trimeric Spike proteins was found. | Very small and limited sample and recruitment not clearly described  Assay validated |
| Jungbauer et al.^135^ | Austria | pre-print | Case series | 100 conlavescent plasma samples | Neutralising antibody titres | ELISA (anti S and anti RBD) Microneutralisation assay | Correlation between neutralising antibidy titre and ELISA was highly significant although quantitatively limited  Severe cases had mean NT50 antibody titer of 1:696, milder cases of 1:208   While increased age and male sex were significantly correlated with neutralising antibody titres, the differences may not be clinically meaningful. | Unclear selection criteria with potential for bias as limited participant characteristics described  Assay validation not clearly described |
| Juno et al.^114^ | Australia | Pre-Print | Case control | Community participants 41 cases, 27 controls median age 59 57% male | IgG, IgM and IgA titres Neutralising ability | ELISA (anti S and anti RBD IgG and IgM and IgA) Microneutralisation | Antibody responses against S and RBD consistently elicited in SARS-CoV-2 infected individuals, the endpoints titres of which correlate significantly with neutralising activity and ACE2 binding inhibition  High plasma neutralisation activity was associated with increased spike-specific antibody, but notably also with the relative distribution of spike-specific cTFH subsets. Expanded populations of spike-specificmemory B cells and circulating Tfollicular helper cells(cTFH)were detected within convalescent donors, while responses to the receptor binding domain (RBD) constitute a minor fraction   Overall, SARS-CoV-2 infection efficiently elicits both S- and RBD-specific B cells in most subjects after recovery, which constitute a 'significant' proportion of the memory B cell pool, which are mostly IgG+ and of a resting memory phenotype    The majority of spike+RBD- class-switched B cells were IgG+ with smaller proportions displaying IgM+ and IgA+ | Unclear inclusion and exclusion criteria  Use of controls although selection unclear with risk of bias  Assay validated |
| Kellam and Barclay^9^ | Multiple populations | Peer Reviewed paper | narrative Review | Multiple populations Inclusion of studies not described | Antibody kinetics | Assays not described in detail | Seroconversion appears to occur in majority of cases before 21 days post symptom onset, and amongst those who do seroconvert, mostly between 10-15 days , with those affected by severe disease appearing to seroconvert earlier and have higher proportions of neutralizing antibodies. A range of titres being described between patients  Early studies suggest that the immune response to SARS-CoV-2 is similar to that for SARS-CoV and MERS-CoV. SARS-CoV-2 seroconversion occurs on a time course that is consistent with other epidemic CoVs and antibodies to spike RBD were the most reliable for case counting in this study. At 2weeks post-symptom onset, antibody titres were statistically higher in critical compared to non-critical patients.  One study exposed Rhesus Macaques with detectable to SARS anti-spike antibodies 28 days from primary infection and neither became infected. | Non-systematic methodology used, lack of clarity on how studies included and lack of quality assessment introduces risk of bias for selection and interpretation of studies |
| Klimstra et al.^118^ | USA | pre-print | Basic Science | 3 convalescent plasma samples | Neutralising antibody titres | IFA PRNT80 | For all patients the first serum sample (ranging from 4-9 days after first reported symptoms) failed to neutralize SARS-CoV-2, however seroconversion occurred in all patients the subsequent day and all samples neutralized  Mean PRNT 80 appears to increase over time | Very small sample and lack of controls limits findings and characteristics of participants not described  Assay validated with control although not clearly described |
| Kohmer et al.^16^ | Germany | Peer Reviewed paper | Case series | Hospitalised participants 33 cases | IgG kinetics | FLA (FaStep) ELISA (Euroimmun IgG, Vircell IgG) IFA PRNT | Between 5-9 days post symptom onset between 58.8% and 76.5% patients had positive assays, variable by assay. Between 10-18 days, between 93.8% and 100% of patients had positive assays (variable by assay)  All but one participant demonstrated neutralizing ability | Lack of clarity on recruitment of participants  Risk of selection bias, particularly as participant characteristics poorly described  Assay validated |
| Kreer et al.^146^ | Germany | pre-print | Cohort | Mixed hospitalised and community participants 12 cases, 48 controls | IgG and IgM kinetics and B cell response | ELISA (IgG) (Euroimmun) Neutralisation assay | A polyclonal B cell response against the SARS-CoV-2 S protein was initiated in all 7 studied cases. A SARS-CoV-2-reactive IgG+ B cell response readily develops after infection with the same B cell clones detectable over time and a preference for facilitating the VH gene segment 3-30. A positive correlation between neutralization and binding could be detected  27 out of 28 neutralizing antibodies found displayed binding to the RBD, but only 31% of the non- neutralizing antibodies. 4 of 28 showed signs of autoreactivity. The development of a neutralizing antibody response is followed by limited additional somatic mutation.  All 48 naive repertoires included at least one κ and one λ chain precursors, with 3 potent neutralising antibodies identified. | Small sample size and selection criteria not well described  Assay validated with controls and used according to manufacturers instructions |
| Laing et al.^82^ | UK | Pre-Print | Cohort | Hospitalised participants 63 cases, 55 controls median age 61 (cases) 36 (controls) | IgG and IgM kinetics | ELISA and LIPS | Most cases produced SARS-CoV-2-specific antibodies, whose host-protective potentials were inferred from strong correlations of RBD-specific IgG. There was no clear correlation of disease severity with either SARS-Cov-2-specific IgM or IgG | Methodology appropriate and well described, although some clarity lacking in selection of participants  Limited compartor group  Assays validated |
| Lee et al.^31^ | China | Peer reviewed paper | Case series | Hospitalised participants 14 cases, 28 controls | IgM and IgG kinetics | LFIA (IgG and IgM) (Alltest) | All symptomatic COVID-19 patients were positive for IgG (n=6/6) and less for IgM (n=4/6). Earliest IgM and IgG detected on day 5. IgG antibodies were absent in 3 cases in the asymp/mild group with positive RT-PCR. IgM was negative in all aymptomatic/mild group with RT-PCR confirmed diagnosis. | Participant selection not detailed  No controls included  Unclear if assay validated |
| Li et al.^102^ | China | Peer reviewed paper | Cohort | Hospitalised particpants 127 cases (all children) | Lymphocyte subsets, immunoglobulin titres, cytokine and inflammatory factor levels | Immunofixation electrophoresis (BNII, Siemens) | 12.6% low IgA, 8.4% low IgG and 5.9% low IgM No controls Low IgA associated with pneumonia OR=4.00 95%CI = 1.13-14.18 (p=0.03) (IgG and IgM not significant) | No ethical procedures detailed  Assay used not stated  Validity limited by small sample size |
| Li et al.^43^ | China | Pre-print | Cohort | Hospitalised participants 1,850 confirmed adult patients | Total antibodies and S-, RBD-, and nucleoprotein (N)-specific IgM and IgG levels over time | CLIA (anti S, anti N, anti RBD IgG and IgM) (Shenzen YHLO Biotech Co.) | Total IgM low in 1st week POS, peaked in 5th week, then decreased to initial levels.  Total IgG peaked in 7th week POS then remained high until end of observation at week 12. Mild/moderate patient groups have earlier S-, RBD-, and N-specific IgM and IgG response and severe/critical patient groups have later higher response (statistically significant) Older patients have higher S-, RBD-, and N-specific IgG response, (statistically significant) | Inclusion / exclusion criteria not described for participant selection  Background of study participants not described  Unlcear if assay validated |
| Liu et al.^119^ | China | Peer reviewed paper | Case series | Hospitalised participants 9 children | Neutralising antibody titres over time and correlates with viral shedding | Not stated | 3/8 cases induced NAbs in the acute phase, 8/9 cases produced medium to high NAb titre in convalescent phase of infection.  Viral shedding persisted after NAb production.  Median NAb titre IC50 = 1,483.9 (307.2–5,925.4) | Participant selection unclear  Sudy design (cohort) limits the validity of results  Assay described and validated |
| Liu et al.^65^ | China | Peer reviewed paper | Cohort | Hospitalised participants 192 cases | RBD specific IgM or total IgM/IgA/IgG levels over time and associated with disease severity | MCLIA (anti RBD IgM and total Ab) (Wantai) | Both total antibody and RBD specific IgM higher in severe than mild cases after day 6 POS (statistically significant) Mean IgM titre peaked at day 21 in severe cases, and positive rate at days 7 to 12 higher in severe cases. No documented peak in IgM for mild cases. | No description of population characteristics  No controls included  Unclear if ELISA assay validated |
| Long et al.^90^ | China | Peer reviewed paper | Case control | Hospitalised participants 37 cases (symptomatic), 37 controls (asymptomatic cases) | IgG and IgM titre | MCLIA (IgG and IgM) (Chonqing Bioscience) | IgG levels in the asymptomatic group were significantly lower (P= 0.005) relative to the symptomatic group in the acute phase.  Change in neutralising Ab response also noted into the early convalescent phase: Median percentage of decrease in NAb titers was 8.3% in the asymptomatic group and 11.7% in the symptomatic group. | Inclusion/exclusion criteria for study participants not explicit  Assays clearly described  Controls used to validate testing |
| Lou et al.^15^ | China | Peer reviewed paper | Cohort | Hospitalised participants 80 cases, 300 controls | Levels of total Ab, IgG and IgM positivity over time and comparison with disease severity | ELISA and LFIA (IgG, IgM and total Ab) (Beijing Wantai Biological Pharmacy Enterprise) CLIA (Xiamen InnoDx Biotech) | Seroconversion rates for total Ab, IgM and IgG were 98.8%, 93.8% and 93.8%, respectively Seroconversion sequentially for total Ab (9 days), IgM (10 days) and IgG (12 days) Seroconversion rate for total Ab and IgM reached 100% and IgG reached 97.1% on days 16, 21 and 29 post symptom onset, respectively | Well conducted although potential for selection bias as process for participant selection not clear  Ethics procedures not described  Unclear if assays were validated |
| Lou et al.^120^ | China | Pre-print | Basic Science | Hospitalised participants 18 cases, 3 controls | Anti-RBD IgG response in convalescent plasma | ELISA (anti RBD IgG) | Plasma from 17 of 18 included convalscent cases demonstrated a high degree of (IgG) binding affinity to SARS-CoV-2 RBD compared to controls | Participant selection not described.  No controls limits validity.  Unclear if assay validated. |
| Lu et al.^121^ | China | Pre-print | Case control | Hospitalised participants 87 cases, 150 controls | Neutralising antibody titres | Microneutralization assay | For samples collected median 37 days (range 23-47 days) post illness onset (n=59) (98.3%) cases developed NAbs with a titre >4, ranging from 4 to >102 | Unclear timing of sampling limits reliability.  Participant selection not stated.  Limited details on assay used and whether validated |
| Luchsinger et al.^110^ | USA | Pre-print | Case control | 370 cases, 10 controls Median age 41 55% female | Neutralising activity of convalescent plasma (NT50 titre) | LFA and ELISA (in house) Neutralisation assay | 83·1% and 92·7% of the CP donor samples had detectable neutralization activity using HIV-S and VSV-S assays, respectively CP donors 2-3 weeks POS had a statistically significant increase in NT50 values compared to CP donors >3 week POS NT50 values of male CP donor samples were ~1·7-fold higher than those from female CP donors with a strong correlation between antibody titre and neutralising response | Participant selection process not stated.  Validity limited by small sample size.  Assay described and validated |
| Lv et al.^158^ | China | Peer reviewed paper | Case control | Hospitalised participants 15 cases, 8 controls age range 37-73 50% female | Plasma binding to SARS-CoV-2 spike, RBD and S2 (OD450 titre) Neutralising activity of convalescent plasma (neutralising titre) | ELISA (anti RBD, anti S2) (in house) icroneutralisation assay | Cross-reactive antibody-binding responses to both SARS-CoV-2 and SARS-CoV S proteins appear to be common but only 1 of 15 patients were able to generate a cross-neutralising response to SARS-CoV-2 and SARS-CoV and this response was weak. | Convenience sampling used and limitation acknowledged.  No controls  ELISA assay validate but unclear if neutralisation assay validated |
| Lynch et al.^39^ | USA | Pre-print | Cohort | Mixed hospialised and community cases 94 cases median age 49, 68% male | Longitudinal IgG and IgM Ab titres | Pylon automated immunoassay (IgG and IgM) | 44/52 (84.6%) and 42/52 (80.8%) had observed IgM and IgG seroconversion at a median of 8 and 10 days, respectively.  Peak measurements were significantly higher for patients admitted to the intensive care unit for all time intervals between 6 and 20 days for IgM, and all intervals after 5 days for IgG. | Sampling approach of participants not clear.  Controls sought and used.  Unclear if ELISA was validated / in-house. |
| Ma et al.^23^ | China | Peer Reviewed paper | Cohort | Hospitalised participants 87 cases median age 48 | IgA, IgG and IgM response | CLIA (IgG, IgM and IgA) (Kaeser 1000) | RBD-specific IgA peaked before IgG and both remained high at day 41 Serum IgM and IgG levels in moderate and severe COVID-19 patients were significantly higher than mild cases, no significant difference was observed between severe and moderate patients.  IgA levels in severe cases were significantly higher than those mild or moderate cases. | Convience sampling of participants.  Controls sought and used.  Unclear if assay validated. |
| Madariaga et al.^91^ | USA | Pre-print | Non-randomised controlled trial | Hospitalised participants 103 cases | Antibody titres and correlates | ELISA (anti RBD, anti S) | Higher anti-RBD and anti-spike antibody titers were associated with plasma donors who were older, male, had higher BMI, had fever and had been hospitalized (p<0.05). | Selection of participants unclear.  Unclear if assays are validated. |
| Marcos-Jimenez et al.^72^ | Spain | Pre-print | Cohort | Hospitalised participants 276 cases, 19 controls | Differential T cell and Ig levels over time and by severity | Immunonephelometry (Immage800) | At admission, IgA and IgM are similar in cases and controls IgG decreased in cases compared to controls (correlated with number of plasma cells) (statistically significant) 10 days after admission IgG decreased further in severe cases (n=37) (statistically significant) | Unclear sampling of participants.  No controls sought.  Assay described and validated. |
| Minervina et al.^52^ | Russia | Pre-print | Basic Science | Community participants 2 cases 50% female | Presence of RBD antibody over time, fraction of reactive CD4 and CD8 T cells over time | ELISA (anti RBD) (National Research Centre for Hematology) | Both donors developed IgG response by day 30 and remained positive to end of follow up (day 45) | Clearly described methods.  In-house assay used, unclear if validated. |
| Mueller et al.^122^ | Germany | Pre-print | Cohort | Community participants 42 contacts of a confirmed case | Neutralising antibody titres and detection of IgG | ELISA (Euroimmun, Roche, Abbott and DiaSorin) Neutralisation assay | PCR confirmed cases had neutralising antubody titres higher than asymptomatic individuals (p<0.01).  Controls had no neutralising antbodies (PCR confirmed and symptomatic) (p<0.001) NAb titre range 1:40 - 1:10240 for PCR The neutralisation titre correlated strongly with all S Ab tests | Limited by population studied (anmal).  Clear description of methodology.  Assay described and validated. |
| Ng et al.^111^ | USA | Pre-print | Cohort | Mixed hospital and community participants 387 cases, 1000 controls | Seroconversion detection rate (IgG and IgM) and neutralising antibody titre | ELISA (IgG and IgM) (Abbott Architect) Neutralisation assay | Neutralising antibody increased concommitantly with IgG and IgM NAb titre mean rose to 1:2048 day 22 | Results aggregated at a late stage limits validity.  Assay described and validated. |
| Ng et al.^159^ | UK | Pre-print | Case control | Hospitalised participants 170 cases, 95 controls | Antibody characteristics of patients with different combinations of HCOV, COV-2 and no prior COV infection Neutralising activity of antibodies from these patient groups | ELISA | Approximately 10% of participants with recent HCoV infections display IgG cross-reactivity towards the Sars-CoV-2 S protein, which also display neutralising potential against SARS-CoV-2 pseudovirus. HCoV patient sera also variably reacted with SARS-CoV-2 S and nucleocapsid (N), but not with the S1 subunit or the receptor binding domain (RBD) of standard enzyme immunoassays. | Numbers of participants in each cohort unclear.  Unclear selectin of participants.  Assays described and validated. |
| Ni et al.^139^ | China | Peer reviewed paper | Case control | Hospitalised participants 14 cases, 6 controls median age 36 57% female | Longitudinal IgG and IgM titres associated with time since discharge  Neutralising antibody titres | ELISA (IgG and IgM) (MABTECH AB, Sweden) | Patients mounted IgG and IgM responses to SARS-CoV-2 NP and S-RBD proteins, and maintained IgG levels for at least 2 weeks post discharge.  Significant correlation between neutralizing antibody titers and anti-S-RBD IgG, but not of anti-NP IgG. | Description of demographics of each cohort unclear.  Unclear how each cohort was recruited.  Multiple assays used, unclear if all validated |
| Okba et al.^67^ | France and Germany | Peer reviewed paper | Cohort | Hospitalised participants 12 cases | Presence/absence of SARS-CoV-2 neutralizing, spike protein–specific, and nucleocapsid-specific antibodies | ELISA (Euroimmun) | After infection, all 3 patients seroconverted between days 13 and 21 after onset of disease. 2 mild and 1 severe case - all seroconverted, earlier seroconversion for severe case compared to mild cases | Clearly described methology.  Validity limited by small sample size.  Assays described and validated. |
| Ou et al.^34^ | China | Pre-print | Cohort | Hospitalsed participants 192 cases: median age 52（IQR, 36-62 years) years and 64% female 130 controls: median age 49 62% female | IgM and IgG and correlates | ELISA (IgG and IgM) (MAGLUMI) and CLIA | Average time of IgM seroconversion was 5-10 days POS, levels peaked 2-3 weeks POS.  IgG peak was around 3-4 weeks POS on average. IgM levels returned to below threshold in around 8 weeks. IgG decline was slower and >90% of patients in this study maintained positive IgG levels at 8 weeks POS. Concentration of IgM in COVID-19 patients was related to gender and disease severity (P <0.01), and the concentration of IgG was related to age and disease severity (P <0.001) | Article selection process not described.  Does not assess whether assays described are validated. |
| Ozturk et al.^66^ | UK | Pre-print | Cohort | Hospitalised pariticpants 32 cases | IgG levels | ELISA (IgG) | IgG levels were very elevated within days of symptoms for hospitalised patients. Milder forms of COVID-19 reached similar lgG levels at 28 days post symptom onset. | Clearly described methodology.  Sampled large proportion of eligible participants.  Assay described and validated |
| Padoan et al.^60^ | Italy | Peer reviewed paper | Cohort | Hospitalised participants 70 cases | IgM, IgA and IgG levels, over time | CLIA (MAGLUMI) ELISA (Euroimmun) | Average levels of IgM and IgA antibodies increased for 6–8 days from the onset of COVID-19. Compared to IgM-Ab, IgA-Ab showed persistently higher levels for the whole observation period, with a peak level at 20–22 days. IgM-Ab levels peaked at 10–12 days and significantly declined after 18 days. | Well described methodology.  Validity limited by convenience sampling.  Assay described and validated |
| Payne et al.^107^ | USA | Report | Cohort | Community participants 382 cases Median age 30 75% male | Seroprevalence Detection of neutralising antibodies | ELISA (anti S) (CDC developed) Microneutralistion assay | 60% participants had a positive ELISA result, and among those 60% also had a positive microneutralization result  Hispanic/Latino participants more likely to have positive microneutralization resultsnon-Hispanic/Latino or unspecified ethnicity participants  Among symptomatic participants with positive ELISA results and positive microneutralization test results a median of 22 days had elapsed since symptom onset at the time of specimen collection  Among 12 participants with positive ELISA results >40 days after symptom onset, eight maintained positive microneutralization test results, including two participants who were tested >3 months after symptom onset. | Methodology clearly described.  Convenience sample with risk of bias.  Unclear if or how assays validated. |
| Percivalle et al.^136^ | Italy | Peer reviewed paper | Cohort | Mixed hospitalised and community participants 390 blood donors, 38 convalescent cases age range 19-70 | Neutralization titers of convalescent patients vs blood donors | Microneutralisation assay | The majority of blood donors had lower neutralising titers than convalescent patients suggesting that neutralizing titers are related to severity of disease.  No statistical anaylsis. | Search strategy and selection of papers not described.  Does not detail if assays used are validated. |
| Perera et al.^76^ | China | Peer reviewed paper | Cohort | Hospitalised and community participants 24 cases, 200 controls, 19 casesof other CoV | Antibody response and disease severity | ELISA (Chondrex Inc, Redmon, US)  Microneutralisation assay | Neutralisation titres and ELISA IgM OD (point biserial correlation coefficient=0.08; p value=0.69) were not correlated with disease severity but severe/critical cases had higher serum ELISA IgG OD than the mild/moderate cases (point biserial correlation coefficient = 0.37; p-value =0.049). | Participant selection criteria not clear.  Multiple time points used for follow up of response.  Some assays modified in-house |
| Premkumar et al.^125^ | USA | Peer reviewed paper | Cohort | Mixed hospitalised and community participants 50 cases | Relationship between RBD binding activity and neutralisation antibody response | ELISA (in house) Neutralisation assay | Total RBD binding IgG correlated with the levels of neutralising anitbodies p=<0.0001.  Those with highest IgM had highest neutralising titers in early convalescence (<6weeks POS) (p=<0.00001). Neutralising antibody kinetics mirrored kinetics of RBD antibody development. No patients had detectable neutralising antibodies within first 8 days. | Participant selection criteria not clear.  Small sample numbers.  Assay described but unclear if validated |
| Prevost et al.^51^ | Canada | Pre-print | Case control | 98 cases median age 56 56% female | Cross reactivity with other HCoV and virus neutralisation | ELISA Neutralisation assay | Cross reactivity with sarbecovirus and betacoronavirus but not alphacoronavirus. 75% elicited antibody response within 2 weeks and all convalescent cases were seropositive Neutralisation capacity decreses signficantly after 6 weeks of symptom onse | No fixed time points for measurement of antibody response.  Selection of cases not clear.  Assay validated and described |
| Qin et al.^81^ | China | Peer reviewed paper | Case control | Hospitalised participants 452 cases median age 58 52% male | Antibody response according to severity | Phorbol 12-Myristate 13-Acetate (PMA)/ionomycin-stimulated lymphocyte function assay | There were no significant differences in the levels of IgA, IgG, between the mild and severe groups, while IgM was slightly decreased in severe cases. | No statistical analysis of data  Validity limited by small sample size.  Assay described and validated |
| Qu et al.^26^ | China | Peer reviewed paper | Cohort | Hospitalised participants 41 cases: median age 62 66% female 38 controls: median age 34 | IgM and IgG levels, over time | CLIA (YHLO) | Seroconversion time of IgG antibody was earlier than that of IgM antibody (12.45±4.36 vs. 13.75±4.60 days, p=0.0019).  97.6% of patients (40/41) were positive with IgG and 87.8% of patients (36/41) were positive with IgM IgG response of the critical group was significantly stronger than that of non-critical groups within 4 weeks POS (p=0.0001). For IgM, the fitting curve of the critical group rose above the cut-off value on day 10, peaked on day 23, then began to decline. | Participant selection process unclear.  No controls sought.  Assay described but unclear if validated |
| Reifer et al.^79^ | USA | Pre-print | Cohort | Community participants 240 cases | IgG response by disease severity | Anti S1, anti S2 IgG (Liaison) | SARS-CoV-2 IgG antibody levels are positively correlated with symptom severity (p-value < 0.01). This trend is true regardless of whether the patient was male or female. | Clear methodology.  Did not include healthy controls.  Assay described but unclear if validated |
| Rijkers et al.^77^ | Netherlands | Pre-print | Cohort | Mixed hospitalised and community participants 63 cases | Antibody response by severity of disease | ELISA (Wantai and Euroimmun) Neutralisation assay | Most patients responded for Ig and IgA between day 10 and 15. 100% had detectable antbodies.  Patients admitted to ITU had similar antibody response to patients in general wards i.e. no statistical difference between the two.  In week 1 43% of hospitalised cohort had detectable neutralising antibodies (VNT50). At 21-28 days all patients had detectable neutralising antibodies with a median titer of 226. In non-hospitalised cohort 87% devleoped anitbodies by day 21-28 with a significantly lower titer than severe hospitalised patients (p<0.0001). 33% of the mild patients remained negative for the presence of virus neutralizing anitbodies. | Rationale snd statistical approach of study limited.  No controls sought  Assay details not provided |
| Robbiani et al.^127^ | USA | Peer reviewed paper | Case control | Mixed hospitalised and community participants 157 cases, 8 controls | Neutralising antibodies | ELISA (in house) Neutralisation assay | Most convalescent plasmas do not contain high levels of neutralising activity, but there are some RBD specific antibodies have very potent neutralising activity. 78% tested positive for anti-RBD and 70% anti-S IgG that were at least 2 SD above the control. Level of neutralising activity were generally low with 33% less than 50 and 79% below 1000 (NT50) Anti-RBD and -S IgG correlated strongly with neutralisation activity, as did age duration of symptoms and disease severity.  Significant difference in neutralisation titers between males and females - higher anti-RBD and -S in males. | Timings of sampling unclear.  Participant selection not described.  Assay described but unclear if validated |
| Rogers et al.^142^ | USA | Peer reviewed paper | Basic Science | Animal study (syrian hamsters) 6 hamsters 17 human cases | Mining potent neutralising antibodies and their passive transfer in an animal model | Neutralisation assay | Passive transfer of nAbs provides protection against disease in high-dose SARS-CoV-2 challenge.  Most potent NAb were those aimed at the RBD-A epitope. Used nAbs aimed at RBD-A and S-B epitope to transfer to Syrian Hamsters. Those that received a high dose passive nAb (against RBD-A) had no evidence of weight loss when challenged with SARS-CoV-2. | Clear methodology.  No controls sought.  Unclear which assay used and whether validated |
| Rogers et al.^141^ | USA | Pre-print | Basic Science | Animal study (Syrian hamsters) 6 hamsters 17 human cases | Description of potent nAbs and passive transfer in an animal model | Neutralisation assay | Passive transfer of nAbs provides protection against disease in high-dose SARS-CoV-2 challenge.  Most potent NAb were those aimed at the RBD-A epitope. Used nAbs aimed at RBD-A and S-B epitope to transfer to Syrian Hamsters. Those that received a high dose passive nAb (against RBD-A) had no evidence of weight loss when challenged with SARS-CoV-2. | Participant selection criteria not detailed.  Assay described and validated |
| Rudberg et al.^89^ | Sweden | Pre-print | Case series | Community partticipants 2149 hospital employees | Seroprevalence amongst healthcare workers | Multiplex (in house) | Seroprevalence 19.1% (n=410) amongst healthcare workers Symptoms with the strongest association to seroprevalence were anosmia (OR 28.43; p=2.02*10^-120), ageusia (OR 19.21; p=1.67*10^-99) and fever (OR 6.27; p=4.24*10^-8). | Participant selection methods unclear.  No standardisation of timing of samples.  Unclear if assays used were validated |
| Ryan et al.^129^ | UK | Pre-print | Trial - non randomised | Animal study (ferrets) 6 ferrets | Neutralising antibody activity Re-challenge day 28 post infection | PRNT | NAb titres for ferrets in high and medium dose groups generally increased longitudinally following challenge. Re-challenged ferrets cleared virus in 5 days post exposure and reduced viral shedding was seen in re-challenged ferrets Immune response to virus 'higher' in re-challneged animal compared to naive animals | Inclusion / exclusion criteria not clearly described for participants.  Selection bias due to presentation determined sample for inclusion.  Assays described but unclear if validated |
| Sandri, et al.^95^ | Italy | Pre-print | Cohort | Community participants 3985 cases and controls | IgG response and correlates with age and sex | CLIA (anti S1, anti S2 IgG) (Liaison) | Higher proportion of IgG positivity in females (p=0.03) but magnitude of anitbody response similar in males and females When assessing a difference of IgG plasma levels across age ranges, males between 41 and 50 y/o had significantly higher plasma levels of IgG than younger individuals (21-30 or 31-40). | Inclusion criteria clearly stated.  Potential for bias due to convenience sampling.  Assay described and validated |
| Sarina Yang et al.^59^ | USA | Peer reviewed paper | Case control | Mixed hospitalised and community participants 42 cases, 144 convalescent plasma samples | IgM and IgG levels in emergency department patients and convalescent patients | Pylon automated immunoassay (IgG and IgM) | IgM reached peak levels between days 10 and 20 POS and then declined IgG and total antibody reached a peak in a similar time frame to IgM but then plateaued Antibody levels in convelscent sera varied widely. | Unclear how participants selected.  Characteristics of controls not described.  Assay described and validated |
| Schmidt et al.^93^ | Germany | Peer reviewed paper | Case series | Communtiy participants (healthcare workers) 406 cases and contacts | Seroprevalence amongst healthcare workers | ELISA (Euroimmun) | Prevalence amongst staff was 2.9% Comparisons of seronegative and seropositive groups shwoed there was no difference in medical conditions between groups. | Poor reporting of population characteristics.  Potential bias as only blood donors included.  Assay described and validated. |
| Selva et al.^160^ | Australia | Pre-print | Basic Science | Mixed hospitalised and community participants 19 cases, 155 adult and 89 child controls | Cross reactivity in children and elderly between SARS-CoV-2, SARS-CoV-1, MERS-CoV and HCoV spike and nucleoprotein (NP) | ELISA (anti RBD) (Thermofisher) | Observed distinct cross-reactive coronavirus serological signatures in healthy children compared to elderly.  Children had elevated CoV-specific IgM signatures, whereas elderly had more mature, class-switched CoV-specific IgA and IgG Children induced elevated IgM, while elderly had higher RBD-specific IgA1 responses while no differences in IgG were observed. | Selection of participants unlcear.  No controls sought.  Assay described and validated. |
| Shen et al.^70^ | China | Peer reviewed paper | Case control | Hospitalised participants 58 cases | IgM response and its correlates with disease severity | CGIA (IgM) (ShanghaiOutdo Biotech Co.) | Significant differences can be observed (p< 0.01) in IgM between mild and severe patient groups. The positive rate in the mild group was 38% (13/34), compared to 0% (0/7) in the severe group at ≤3days after symptom onset. At 4–7 days POS, the positive rate of the mild group was 64% (16/25), compared to 16.7% (1/6) in the severe group. At 15–21 days after symptom onset, the positive rate of the mild group was 100% (15/15), compared to 75% (6/8) in the severe group. | Participant selection criteria not stated.  Limited validity due to small population size of convalescent donors.  Assay described but unclear if validated. |
| Siracusano et al.^123^ | Multiple populations | Peer reviewed paper | Narrative review | Multiple populations Inclusion of studies not described | Narrative revew of Ab and nAb response to COVID-19 | N/A | Estimate of development of nAb at day 6 for severe disease and day 8 for mild disease with a sharp accesion day 1 and subsequent plateau | Methodology clear with appropriate statistical testing.  Inclusion and exlcuison criteria of participants not explicit.  Assay described and validated. |
| Solbach et al.^37^ | Germany | Pre-print | Cohort | Community participants 110 cases 55% female | Antibody kinetics (onset, duration) in a low incidence population | ELISA (IgG and IgA) (Euroimmun) | Within a period of 50 days after the infection, 84/110 (76 %) and 78/110 patients (71 %) of the patients developed antibodies for IgA and IgG, respectively In around 30 % of the patients with mild to moderate symptoms, no significant antibodies could be detected in two consecutive analyses No correlation between symptoms and antibody profile or with gender. | Characteristics of participants not described.  No ethical statement.  Assay described and validated. |
| Solodky et al.^86^ | France | Peer reviewed paper | Case series | Community participants 329 cases | Presence of antibody | LFIA (Toda Coronadiag) | 30% of cancer patients with posuitive RT PCR had positive Ab at 15 days post symptom onset, 71% for healthcare workers Rate of seroconversion significantly lower in cancer patients compared to healthcare workers (30% vs 71%, p=0.04) | Potential bias as mild patients were all hospital staff, convenience sampling.  Participant selection criteria not explicit.  Assay described but unclear if validated. |
| Staines et al.^84^ | UK | Pre-print | Cohort | Mixed hospitalised and community cases 177 cases median age 64 57% male | Seroconversion timing and correlates | ELISA (Omega Diagnostics) | 8.5% (95%CI 5.2% -13.5%: 15/177) did not seroconvert during the entire follow up period, 7.3% (95%CI 4.3% - 12.1%; 13/177) seroconverted after enrolment and 84% (95%CI 78% - 89%; 149/177) had already seroconverted at the time of the first serological test. 2-8.5% of patients may not develop detectable IgG antibody responses to SARS-CoV-2 for weeks following infection. Seroconversion peaks around d20 then plateaus | Clear methodology.  Potential asymptomatic cases included.  Assay described and validated |
| Sun et al.^44^ | China | Peer reviewed paper | Cohort | Hospitalised participants 38 cases | Neutralising Ab titres | ELISA (anti N and anti S IgM and IgG) | N-IgM and S-IgM in some non-ICU patients reached a peak in the second week, while N-IgG and S-IgG continued to increase in the third week. S-IgG was significantly higher in non-ICU patients than in ICU patients in the third week. In contrast, N-IgG was significantly higher in ICU patients than in non-ICU patients | Clear methodology.  Applicability to wider population limited as an animal model.  Assay described, unclear if validated. |
| Suthar et al.^116^ | USA | Pre-print | Case series | Hospitalised participants age range 33-87 59% male | Antibody kinetics and Neutralising Ab titres | Multiple assays (in house) | IgG subclass analysis showed that the COVID-19 patients exclusively made RBD-specific IgG1 and IgG3, with no detectable IgG2 or IgG4. Taken together, these findings illustrate that antibody class-switching to IgG occurs early during acute infection. Antibody responses against the RBD as well as SARS-CoV-2 neutralisation titres can be detected in a majority of patients around day 8 POS. When the number of days after PCR confirmation is used to assess the duration of infection, both RBD-binding titers and viral neutralization titers can be detected in many patients already between days 2-6.  Both RBD-specific and neutralizing antibody responses occur rapidly after SARS-CoV-2 infection. | Potential bias as 85% female participants.  Inclusion / exclusion criteria for participants not clear.  Assay described and validated. |
| Tan et al.^42^ | China | Pre-print | Cohort | Hospitalised participants 66 cases (3 children) median age 49 52% male | IgM and IgG antibody titres | ELISA (IgG and IgM) (Livzon Diagnostics) | The anti-nucleocapsid-protein IgM started on day 7 and the positive rate peaked on day 28, while that of IgG was on day 10 and day 49 POS. In severe patients, IgM and IgG appear earlier, and their titers are significantly higher than non-severe patients (p<0.05) The positive rate for IgM increased to 28 days (57.1%) then decreased to 33.3 % at 42 days. The positive rate for IgG increased to 74.3 % at 28 days and increased to 86.7% at 42 days, and persisted | Selection of particiants unclear.  Limited patient numbers included in follow up.  Assay described but unclear if validated. |
| Thompson et al.^140^ | UK | Pre-print | Cohort | Community participants 1000 unknown COVID-19 status blood donors, 100 controls | Neutralising antibody titres | ELISA (in house) Neutralisation assay | In 6 positive control samples (samples taken from control population during pandemic ie not pre-pandemic controls) neutralising antibody IC50 range 83.18- 323.59; R2 range: 0.79-0.96.  In 5 blood donor samples IC50 range 78.42-3434.90; R2 range: 0.64-0.95 Presence of neutralising antibodies correlates with presence of IgG/M on ELISA - ELISA OD correlates with IC50 on pMN R2 = 0.49, p=0.036 | Limited to only participants with moderate and severe disease limits validity.  Inclusion and exclusn criteria for participants not clear.  Assay described and validated |
| To et al.^156^ | France | Peer reviewed paper | Cohort | Hospitalised participants 23 cases median age 62 57% male | IgG and IgM titres | EIA (in house) | Serum antibody levels did not correlate with clinical severity More patients had earlier seropositivity for anti-RBD than anti-NP for both IgG (43% RBD earlier vs 9% NP earlier) and IgM (26% RBD earlier vs 17% NP earlier). More patients had earlier seroconversion for IgG than IgM for anti-NP (IgG earlier 26% vs IgM earlier 4%) and anti-RBD (IgG earlier 57% vs IgM earlier 4%).  Seropositivity for anti-NP IgG 94% (n=16) by 14 days, anti-NP IgM 88% (n=14), anti-RBD IgG 100% (n=16), 94% anti-RBD IgM (n=15) | Clear metholodology.  Unclear how controls sought.  Assays described and validated |
| Vabret et al.^17^ | Multiple populations | Peer reviewed paper | Narrative review | Multiple populations Inclusion of studies not described | Narrative review on immunity to COVID-19 | N/A | Robust B cell response of virus-specific IgM, IgG and IgA, and nAbs in the days following infection.  IgG specific to SARS-CoV-2 trimeric spike protein was detectable in serum up to 60 days after symptom onset, but IgG titers began decreasing by 8 weeks post symptom onset The SARS-CoV-1 humoral response is relatively short lived, and memory B cells may disappear altogether, suggesting that immunity with SARS-CoV-2 may wane 1–2 years after primary infection. | Potential for bias as population self-selected for presentation.  Assay described and validated |
| Varnaite et al.^138^ | Sweden | Pre-print | Case control | Hospitalised participants 20 cases, 7 controls | Antibody kinetics, neutralising antibody kinetics, T-cell response | ELISA (anti S1 IgG) (Euroimmun) IFA Microneutralisation assay | IgA, IgG and IgM antibody levels positively correlated with neutralizing antibody titers Total and spike S1-specific SARS-CoV-2 IgG antibody level positively correlated with the number of days since symptom onset (rs= 0.577, P =0.01 and rs= 0.603, P =0.005 respectively Total SARS-CoV-2-specific IgG antibody levels positively correlated with neutralizing antibody titers (rs= 0.865, P <0.001) | Methology clearly stated.  No controls sought.  Assay described and validated. |
| Vogelzang et al.^54^ | Netherlands | Pre-print | Case control | Mixed hospitalised and community participants 284 cases median age 31 | Antibody response in non-hospitalised and hospitalised patients | Multiple in-house assays | Antibodies detected in both hospitalised and non-hospitalised groups Non-significant trend in declining IgM and IgA levels as time progresses in both hospitalised and non-hospitalised Non hospitalised patients have a slower rise in IgG and has a weaker response than hospitalised patients In the group highly sustected of having COVID-19 9 of 14 patients seroconverted for IgG within 15 days POS and 3 seroconverted after | No ethical statement provided.  No controls sought.  Assay described and validated |
| Wang et al.^151^ | China | Pre-print | Case series | Animal study (rabbits) 10 human cases | Neutralising antibody kinetics | Neutralisation assay | Potential neutralising epitopes (involved in RBD) identified in 2 cases (IgG) and 1 case (IgM) Cross reactivity of SARS-CoV-1 Ab to SARS-CoV-2 in rabbits | Selection of participants not described.  Methodology clearly described.  Assay described and validated. |
| Wang et al.^25^ | China | Peer reviewed paper | Cohort | Hospitalised participants 26 cases age range 5-72 | Antibody kinetics | CGIA (Innovita) | IgM and IgG detected from day 7 POS. All 25/26 developed IgG but 11/26 cases did not develop IgM. One case had not developed antibodies by day 66 post onset (5 year old female) All other cases maintained IgG until the end of their follow up (range 16-39 days) | Methods clearly stated.  Assay described and validated. |
| Wang et al.^134^ | China | Peer reviewed paper | Cohort | Hospitalised participants 70 cases mean age 45 59% female | Neutralising Ab titres | In house assay | The mean titre of neutralizing antibodies (average: 1:271.2) was the highest at 31-40 days since onset and antibody titers at 31-40 days were significantly higher than 10-20 days POS.  The antibody levels according to different time course since onset were significantly different (P=0.0012). The effect of gender was not statistically significant.  A significant neutralizing antibody response was observed in older patients with a geometric mean titer of 1:220.1 (95% CI, 71.8 to 674.8) compared to patients at age of 16-30 (1:71.0, 95% CI, 27.7 to 181.8) and at age of 31-60 (1:200.6, 95% CI, 150.4 to 267.6) (P=0.0140). | Criteria for participant selection not clearly described.  Assay described and validated. |
| Wang et al.^137^ | USA | Pre-print | Case control | Hospitalised participants 35 cases 50% female | Neutralisation activity in severe vs non severe patients. | In-house neutralisation assay | Titers of antibodies binding to the viral nucelocapsid and spike proteins were significantly higher in patients with severe disease Mean plasma titer was 8 fold higher in severe group (p=0.036) for NP directed antibodies and for S directed antibodies (p=0.016) Anitbody neutralisation titers against the pseudovirus and live virus were higher in the sicker patients | Study powered towards both validating assay and studying antibody kinetics.  Participant selection criteria not stated.  Assay described and validated within study |
| Wang et al.^45^ | China | Pre-print | Case control | Hospitalised participants 116 cases |  | CLIA (IgG and IgM) (YHLO) | IgM levels peaked in 4th week after onset of COVID-19 pneumonia, while serum IgG levels increased over 8 weeks. Serum IgM was higher in deceased patients than mild-moderate patients (p=0.019), but not IgG.  Serum IgM levels were positively correlated with IgG levels (r=0.279, p=0.031), and negatively correlated with clinical outcome (r= 0.337, p= 0.008) No significant correlations between IgM levels and sex, comorbidities or duration between onset and antibody detection in patients with COVID-19 pneumonia - unmatched and when matched on age | Clear methodology.  Selection of participants not clear.  Assay described and validated. |
| Wec et al.^148^ | Animal study | Pre-print | Basic Science | Animal study | Neutralising activity of cross reacting SARS-CoV-1 Ab to SARS-CoV-2 | In house assay | 0.14 % of class-switched MBCs were SARS-CoV-2 S-reactive (3 fold higher than control) 9 out of 200 antibodies displayed neutralizing activity at the 100 nM concentration tested, 8 of which targeted RBD and 1 targeted NTD  IC50s of the RBD-directed nAbs ranged from 0.05-1.4μg/ml against SARS-CoV-2 | Paticipant selection criteria not described.  No detail on what constituted 'severe disease' and admission to ITU limits applicability.  Assay described and validated. |
| Wu et al.^133^ | China | Pre-print | Cohort | Hospitalised participants 175 cases median age 50 53% female | Neutralising activity of SARS-CoV-2 | ELISA (in house) Neutralisation assay | Some cross-binding Ab to SARS-CoV-1 but not cross-neutralising Ab NAbs titers moderately correlated with spike binding antibodies targeting RBD (r=0.51, p<0.0001), S1 (r=0.42, p<0.0001), and S2 (r=0.435, p<0.0001) - n=175 30% of recovered patients had low NAb titres (ID<500). NAb titers of elderly and middle-age recovered patients were significantly higher than those of young recovered patients p<0.0001. | Clear methodology.  Methods not fully described.  Assay described and validated. |
| Wu et al.^147^ | Lab based | Peer reviewed paper | Basic Science | 1 case | Neutralising activity of SARS-CoV-2 | In house assay | Details of specific neutralising antibodies identified from single donor - 4 Ab identified, median IC50 values ranging from 0.177 to 1.375 mg/ml | Participant selection methods unclear.  No fixed time points for sampling.  Assay decribed but not validated. |
| Xiang et al.^32^ | China | Peer reviewed paper | Cohort | Hospitalised participants 109 cases | IgG and IgM titers | ELISA (IgG and IgM) (Livzon) | IgM cumulative seroconversion increased quickly from the 9th day and IgG from the 11th day POS.  Both antibodies were seropositive in nearly all the patients for more than 30 days IgG and IgM detected as early as day 4.  The seropositive rate of IgG was observed decreased around the 28th POS | Clear methodology.  Potential for bias from retrospective data collection.  Assay described and validated. |
| Xiao et al.^40^ | China | Peer reviewed paper | Case series | Hospitalised participants 34 cases | IgG and IgM titers | CLIA (IgG and IgM) (Shenzhen) | Profile of specific antibodies to SARS-CoV-2 is similar to SARS-CoV.  Detectable and continuous high level IgM indicated acute phase of infection. IgG responded later and persisted. IgG detected in all patients (n=6) tested at 7 weeks after onset | Description of cohorts not clear.  No controls sought.  Assay described but unclear if validated. |
| Xie et al.^62^ | China | Peer reviewed paper | Cohort | Hospitalised participants 56 cases | Antibody and cellular response, clinical characteristics of patients | CLIA (IgG and IgM) (YHLO) | IgG maintains high levels with severe and non-severe disease IgM increased in 1 week and then started to decline week 4-5 | Selection of participants unclear.  Validity limited by small sample suze.  Assay described but unclear if validated. |
| Yongchen et al.^64^ | China | Peer reviewed paper | Cohort | Hospitalised cases 21 cases | IgG and IgM titers | CGIA (Innovita) | All symptomatic patients developed antibody response; an earlier antibody response was seen in patients with severe disease.  IgM was not detected for 6/11 non-severe pts but all (5/5) severe patients were IgM and IgG positive All of 17 symptomatic patients (100%) were seropositive at the time point of discharge or during follow-up period  Only 1 of 5 aysymptomatic cases generated an antibody response at week 3 | Statistical methods unclear.    Selection of particpants not described.  Assay described and validated |
| Zeng et al.^96^ | China | Peer reviewed paper | Cohort | Hospitalised participants 331 cases 67% female median age 42 (F) 45 (M) | IgG concentration by sex and disease severity | CLIA (IgG) (YHLO) | Concentration of IgG antibody in mild, general, and recovering patients showed no difference between male and female patients In severe status, compared with male patients, there were more female patients having a relatively high concentration of IgG antibody Generation of IgG antibody in female patients was stronger than male patients in disease early phase | Clear metholodolgy.  Selection of cases not fully described.  Assay described and validated. |
| Zeng et al.^103^ | China | Peer reviewed paper | Case series | Hospitalised participants 6 infants born to 6 cases | IgG, IgM, IL-6 levels | CLIA (IgG and IgM) (YHLO) | All 6 babies had antibodies detected in serum and were asymptomatic Two infants and their mothers had IgG and IgM concentrations higher than the normal level (<10 AU/mL) Three infants had elevated IgG levels (75.49, 73.19, 51.38 AU/mL) but normal IgM levels; all 3 mothers had elevated IgG and 2 also had elevated IgM levels.  Inflammatory cytokine IL-6 was significantly increased in all infants. | Methods clearly stated.  Demographics of participants not clear.  Assay described and validated. |
| Zhang et al.^30^ | China | Pre-print | Cohort | Hospitalised paritcipants 222 cases median age 62 52% female | IgG, IgM, IL-6 levels | CLIA (IgG and IgM) (Shenzhen) | IgG was first detected on day 4 of illness, and its peak levels occurred in the 4th week, whereas IgM was first detected on day 3 of illness, and its peak levels occurred in the 2nd week Severe cases more commonly found in those with high IgG levels in comparison to the low level group (51.8% vs 32.3%, p=0.008).  Severe cases occured more frequently in those with lower IgM levels (<34.1 AU/ml) than those with high IgM levels (>3.04AU/ml) (81.3% vs 40%; p=0.024) Severe patients had higher IL-2, IL-6 and IL-10 than non-severe patients. Severe patients also had higher proinflammatory cytokines levels including IL-2, IL-6 and IL-10, and decreased CD4+ T cell count (p<0.05). | Limited description of recruitment process.  Unclear if participants representative of wider population.  Assay described and validated. |
| Zhang et al.^61^ | China | Peer reviewed paper | Cohort | Hospitalised pariticpants 122 cases median age 38 71% female | IgG and IgM titres | EIA (IgG and IgM) (Yahuilong Biotechnology) | Fifty-eight (51.79%) were positive for both IgM and IgG, 7 (6.25%) were negative for both antibodies, 1 (0.89%) was positive for only IgM, and 46 (41.07%) were positive for only IgG.  IgM antibody appeared within a week post–disease onset, lasted for 1 month, and gradually decreased, whereas IgG antibody was produced 10 days after infection and lasted for a longer time. | Lacking statistical analysis.  Recruitment of particpants unclear.  Assay described but unclear if validated. |
| Zhang et al.^33^ | China | Peer reviewed paper | Cohort | Hospitalised participants 6 cases age range 30-50 | IgG and IgM titres | ELISA (IgG and IgM) (Livzon) | 5/6 had weakly reactive IgM and 5/6 had high IgG titres Paper focused on the use of convalescent plasma | Limited description of method.  Ethics not detailed.  Assay described and validated. |
| Zhang et al.^48^ | China | Peer reviewed paper |  | Hospitalised participants 6 children age range 7-131 months | Total Ab, IgG and IgM positivity only (no measured titres) | CLIA (total, anti S and anti RBD IgG) (Kehua Bio-Engineering) | Relative quantitative analysis showed that total or IgG antibody for nucleocapsid and spike-RBD protein production were significantly increasing over the days after illness onset The percentage of CD3+, CD4+, and CD8+ T cells between infected and uninfected cases were comparable. | Clear metholodology.  Small sample size of convalescent patients limits validity.  Assay described and validated. |
| Zhang et al.^73^ | China | Pre-print | Cohort | Hospitalised participants | Total antibodies (Ab), IgG, IgA and IgM titres | CMIA | The total sero-positive conversion rate for Ab, IgG, and IgA of asymptomatic patients was 90.9% (20/22), 95.5% (21/22) and 90.9% (20/22), respectively All symptomatic patients experienced sero-positive conversion of total Ab, IgG and IgA.  The total seropositive conversion rate of IgM reached 45.5% (10/22), 62.5% (20/32), and 63.2% (12/19) for asymptomatic, pre-symptomatic, and symptomatic patients, respectively. | Clear metholodology  No controls sought  Assay described and validated |
| Zhang and Qu.^49^ | China | Pre-print | Cohort | 67 cases | Ab neutralisation | ELISA | 51 sera showed cross-neutralization with SARS-CoV-1, 12 (17.91%, 7.46% plus 10.45%) with MERS-CoV, and 7 (10.45%) with both. Compared to non-severe group, severe group exhibited higher titers of anti-S1 (p=0.012) and anti-S2 antibodies (p=0.011) and higher neutralization titers (p=0.001), but no difference for binding avidity nAb response was correlated with the severity of disease, though older age (p<0.001), longer course of disease (p=0.007), more comorbidities (p=0.044), and underlying diseases (p<0.001) were higher in severe group | Limited description of participants.  No description of ethical approval or consent process.  Assay described but unclear if validated. |
| Zhao et al.^24^ | China | Peer reviewed paper | Cohort | Hospitalised participants 137 cases | IgG and IgM titres | ELISA (Wantai) | The seroconversion rate for Ab, IgM and IgG was 93.1% (161/173), 82.7% (143/173) and 64.7% (112/173) respectively  Critical patients showed significantly higher serocoonversion Ab values than non-critical cases in about 2-week after onset (p=0.02) No significant difference on the average seroconversion value of Ab tests between critical and non-critical patients before day 12 2 weeks after onset, critical patients showed significantly higher Ab S/CO values than non-critical cases (p=0.02) and this association was not significant in either IgM or IgG tests | Unclear participant selection methods  Process for control indentification not described  Assay described and validated |
